# Susceptibility of calves fed unpasteurized milk from cows infected with highly pathogenic avian influenza H5N1

**DOI:** 10.1038/s41467-026-76300-1

**Published:** 2026-08-21

**Authors:** Kaitlyn M. Sarlo Davila, Amy L. Baker, Paola M. Boggiatto, Mitchell V. Palmer, Ellie J. Putz, Steven C. Olsen, Giovana Ciacci Zanella, Alessandra Campos, Alexandra C. Buckley, Bailey Arruda

**Affiliations:** 1 Ruminant Diseases and Immunology Research Unit, National Animal Disease Center, Agricultural Research Service, United States Department of Agriculture, Ames, IA USA; 2 Virus and Prion Research Unit, National Animal Disease Center, Agricultural Research Service, United States Department of Agriculture, Ames, IA USA; 3 Infectious Bacterial Diseases Research Unit, National Animal Disease Center, Agricultural Research Service, United States Department of Agriculture, Ames, IA USA

**Keywords:** Influenza virus, Animal physiology, Viral transmission

## Abstract

Highly pathogenic avian influenza (HPAI) H5N1 was confirmed in a dairy cows in Texas March 2024 and milk was identified as a critical source of virus spread within and between dairy herds. This study demonstrates that calves fed infected unpasteurized milk from infected cows became infected, showed clinical signs including nasal discharge and increased respiratory effort, and seroconverted. Live virus was persistently detected in nasal swabs, even after calves were transitioned to milk from non-infected cows. This study also demonstrates the presence of neutralizing antibodies in milk provides from protection from infection as an additional group of calves fed milk from infected cows also shedding neutralizing antibodies did not become infected, show clinical signs, or seroconvert. These data demonstrate that milk diverted from the human food supply in H5N1 positive dairy herds or suspect cows should not be fed to calves without pasteurization, as H5N1 may further adapt to the bovine respiratory tract and the risk of calves as a source of farm to farm transmission has not yet been evaluated. As characterization of HPAI in the dairy community continues the determination of routes of transmission is an essential step to inform subsequent research on intervention and vaccination strategies.

## Abstract (web summary)

In this study, the authors show that highly pathogenic avian influenza virus can be transmitted to calves fed unpasteurized milk from infected cows with infected calves showing clinical signs but that the presence of neutralizing antibodies in milk provides protection from infection.

## Introduction

Highly pathogenic avian influenza (HPAI) H5N1 clade 2.3.4.4b genotype B3.13 was confirmed in a dairy cow in Texas on March 25, 2024, by the US Department of Agriculture (USDA) National Veterinary Services Laboratories in response to a multi-state investigation into milk production losses.^1^ Viruses of the goose/Guangdong lineage in the hemagglutinin clade 2.3.4.4b had previously been detected in the United States (U.S.) in 2021 following widespread dispersion across Asia and Europe. H5N1 viruses in this clade were detected across the U.S. in mortality events in wild bird species and wild mammals^2,3^. Additionally, outbreaks occurred in numerous commercial and backyard poultry premises, leading to culling to control the spread^4^. Subsequently, other outbreaks in dairy cattle were rapidly identified in Texas as well as other U.S. states^5^. As of March 9, 2025, 1088 cases were confirmed in 19 states^6^.

The typical case description of clinical cows includes a decrease in milk production and noticeable clinical signs such as thick yellow milk with flecks and/or clots. Infectious virus and viral RNA have been consistently detected in milk from affected cows^7,8^. The amount and duration of virus shed in milk from the inoculated mammary quarters point to milk as a critical source of virus for potential spread within and between dairy herds^9^. Domestic cats consuming raw milk from affected cows developed fatal systemic influenza infection^8^. Although pasteurization has been shown to inactivate the virus in milk^10^, epidemiological investigations on affected dairy herds demonstrated the presence of viral RNA in milk samples for up to 2 weeks before the appearance of clinical signs and diagnostic confirmation of infection^8^. During this lag, unpasteurized milk may pose a risk to calves. Additionally, some dairies and calf ranches continue to feed unpasteurized non-saleable milk, despite recommendations to pasteurize waste milk fed to calves^11,12^. Here, we sought to determine if the H5N1 genotype B3.13 strain could be transmitted to calves fed unpasteurized milk from virus-positive lactating cows and whether the presence of neutralizing antibodies in milk provided any protection from infection to the calves. This study demonstrates that the H5N1 genotype B3.13 strain could be transmitted to calves fed unpasteurized milk from virus-positive lactating cows, and that calves became infected and showed clinical signs, but that the presence of neutralizing antibodies in milk provides protection from infection, as an additional group of calves fed milk from infected cows that also shed neutralizing antibodies did not become infected or show clinical signs.

## Results

### Virus and neutralizing antibodies in milk fed to calves

To determine if the H5N1 genotype B3.13 strain could be transmitted to calves fed unpasteurized milk from virus-positive lactating cows, we confirmed the presence of viral RNA in milk fed to calves. For Group 1, calves were fed milk from two experimentally inoculated cows during 1 through 4 days post-inoculation (DPI), and viral loads increased after 1 DPI in those cows. To further determine if the presence of neutralizing antibodies in milk provided any protection from infection to Group 2 calves were fed milk from two cows in mid-infection following seroconversion (15–19 DPI), spiked with 187.5 ml of milk from a cow early in infection (3 DPI) to keep viral load as similar as possible to Group One. The 2 cows providing milk to calves in Group Two each had serum HI titers of 1:320 and milk VN titers of 1:40 on 13 DPI before feeding began on 15 DPI.

### Clinical signs of infection in calves

**Fig. 1:**
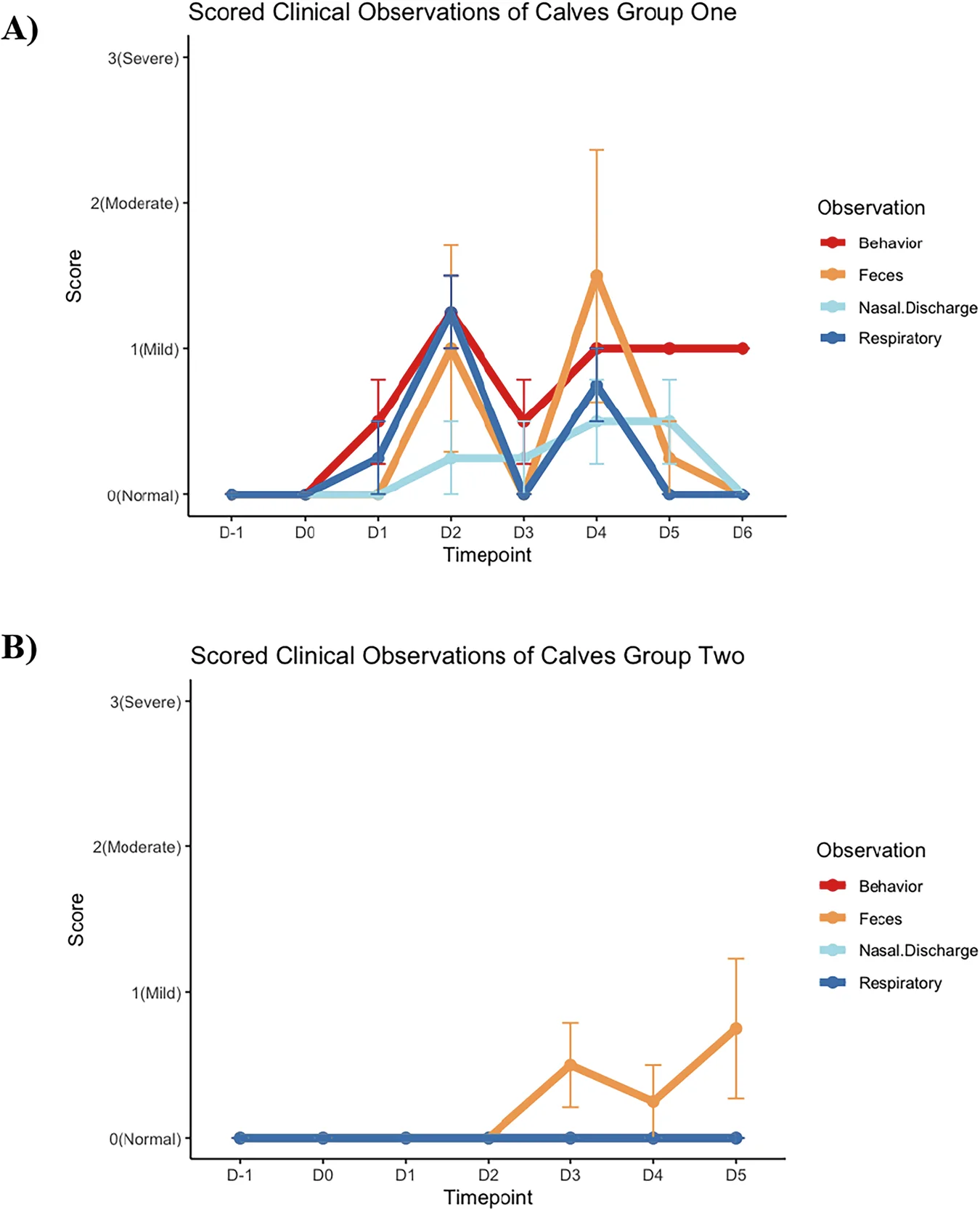
Clinical observations of a group of calves fed milk containing HPAI virus and a second group of calves fed milk containing HPAI virus and antibody. **A** Clinical observations of Group One calves (*n* = 4) fed milk containing the virus. Calves showed clinical signs, including nasal discharge and increased respiratory effort. **B** Clinical observations of Group Two calves (*n* = 4) fed milk containing virus and antibody. Calves did not show clinical signs. Normal behavior, feces, nasal discharge, and respiration were scored as a zero, while abnormal clinical signs were scored one on a one-to-three scale, with one representing the mildest symptoms and three representing the most severe. Full criteria for clinical scores used to assess dairy calves are shown in the Supplementary Information. Behavior clinical signs were scored from mild lethargy (1) to marked lethargy (3). Feces clinical signs were scored from semi-formed (1) to watery (3). Nasal discharge was scored from a small amount (1) to a copious amount (3). Respiration was scored from slightly increased respiratory effort (1) to severe respiratory distress (3). Data are represented as mean values ± SEM. Source data are provided as a Source data file.

Scored clinical observations were used to evaluate the clinical signs of HPAI infection in calves consuming infected milk with and without neutralizing antibodies. Normal behavior, feces, nasal discharge, and respiration were scored as a zero, while clinical signs were scored on a one through three scale, with one representing the mildest symptoms and three representing the most severe. Full criteria for clinical scores used to assess dairy calves are shown in Supplementary Information. The control calf consuming milk from non-inoculated cows did not develop any clinical signs of disease during the study. However, mild lethargy and slightly increased respiratory effort were observed in all 4/4 calves in Group One consuming unpasteurized milk from antibody negative experimentally inoculated cows beginning 1 DPI, as shown by the increased behavior and respiration scores in Fig. 1. By 2 DPI, loose stool and nasal discharge were observed in 3/4 calves, as indicated by the increased feces and nasal discharge scores in Fig. 1. These signs continued intermittently and resolved by 6–7 DPI in inoculated calves. Elevated rectal temperatures, greater than 39.5 °C, were observed beginning on 2 DPI in 2/4 calves, as shown in Table 1, and remained elevated in two calves (2410 and 2414) through 5 DPI, with the highest temperatures (40.6 °C) recorded on 4 DPI. Temperatures were not recorded for Group One on 1 DPI due to thermometer failure and were also not recorded for the two calves necropsied at 6 DPI.

**Table 1:**
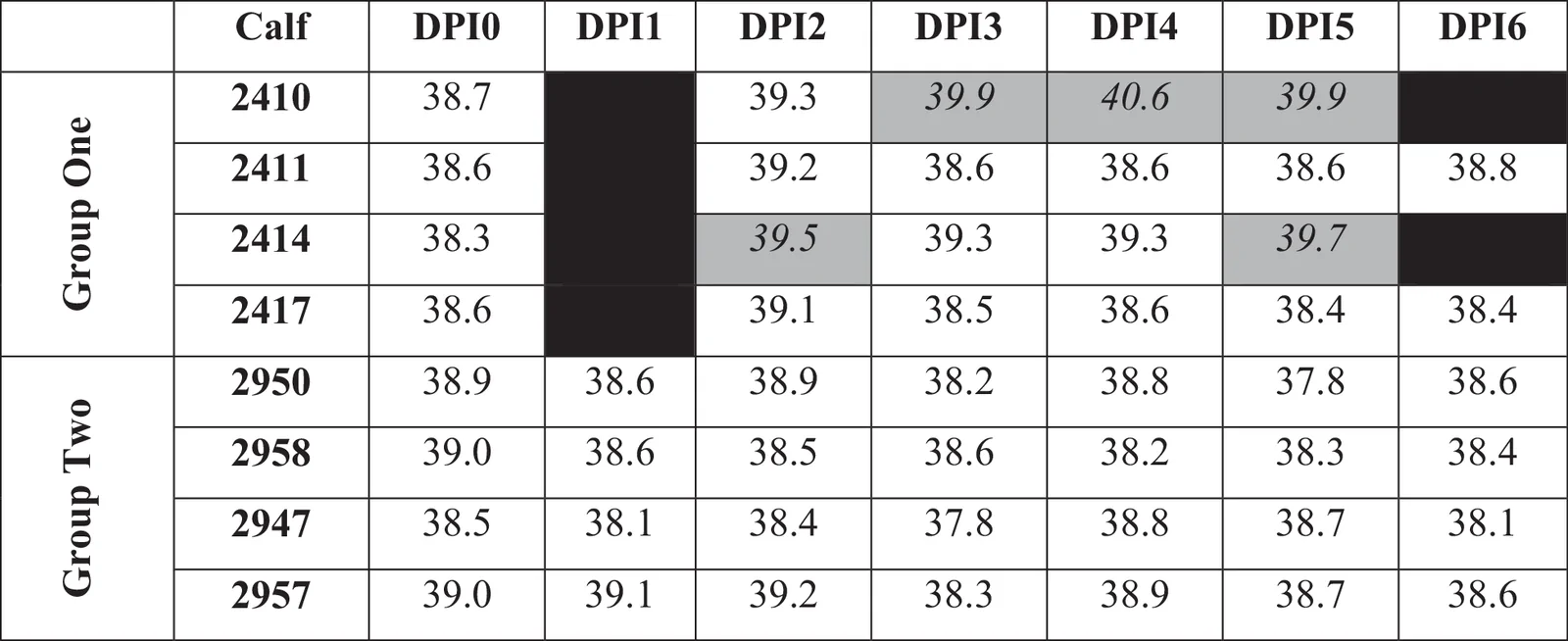
Rectal temperatures of calves fed milk with virus (Group One) and milk with virus and antibody (Group Two)

Fever (rectal temperature >39.5 °C) indicated by gray shading and italics; black indicates not recorded.

**Fig. 2:**
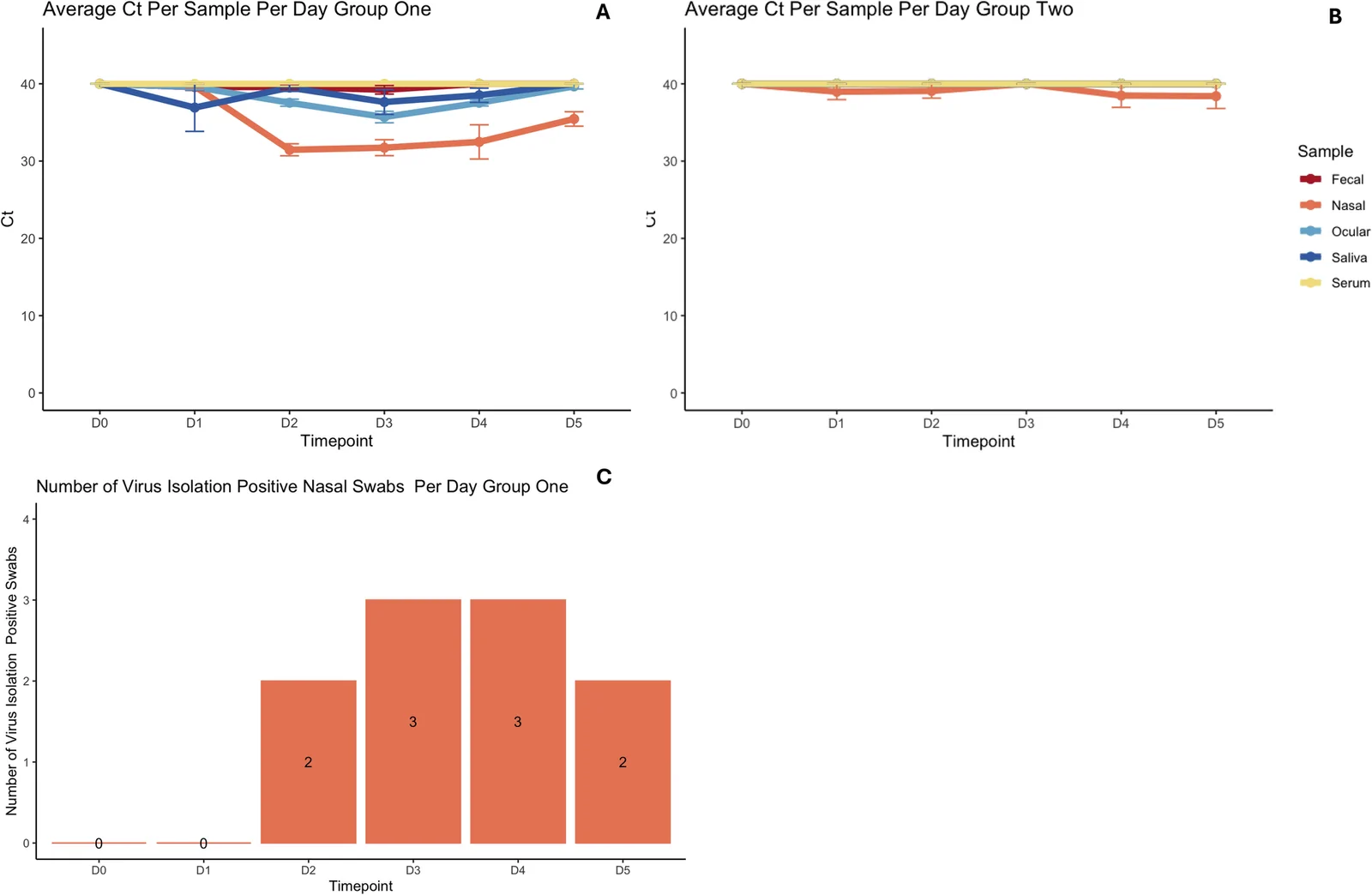
Viral RNA detection in experimental samples. **A** Average Ct values for calves (*n* = 4) in Group One from experimental samples in connected lines. 4/4 calves had viral RNA detected in nasal swabs. **B** Average Ct values for calves (*n* = 4) in Group Two from experimental samples in connected lines. Only 1/4 of calves had viral RNA in nasal swabs. **C** Number of virus isolation positive nasal swabs (*n* = 4 per day) from Group One. Live virus isolation from nasal swabs with detection persisting after calves were transitioned to milk from non-infected cows. Data are represented as mean values ± SEM. Source data are provided as a Source data file.

For Group Two calves fed milk with both virus and neutralizing antibody, no clinical signs were observed other than occasional, intermittent loose stool, as shown by the increased feces scores in Fig. 2. Calf 2958 had loose stool on 5 and 6 DPI, calf 2947 had loose stool on 4 and 6 DPI, and calf 2957 had loose stool on 4 DPI. Elevated rectal temperatures were not observed in any of the calves from Group 2.

### Virus detection

All samples collected from the negative control calf were negative for IAV by reverse transcription quantitative real-time PCR (RT-qPCR). In Group One, calves fed milk containing virus but not antibody, nasal swabs from 4/4 calves were PCR positive (Ct <35) starting on 2 and 3 DPI. The lowest average Ct values (highest viral load) were observed at 2 DPI (Ct 31.5) and 3 DPI (Ct 31.7). There was a strong negative correlation (−0.55) between Ct values from the nasal swabs and behavioral scores in the calves, indicating that as Ct values dropped and viral load increased, calves became more lethargic. Virus isolation was attempted on all nasal swabs, and live virus was isolated from nasal swabs from 2/4 nasal calves on DPI 2, ¾ calves on DPI 3 and 4, and 2/4 calves on DPI 5. Ocular swabs remained in the suspect range for 4/4 calves on DPI 2 and DPI 4. At 3 DPI, one calf (2410) had a PCR-positive ocular swab (Ct = 33.9) while the ocular swabs from the other three calves remained in the suspect range. By 5 DPI, all ocular swabs were negative. No fecal swabs or serum samples were PCR positive at any time point. Only 2/4 saliva samples were PCR positive (Ct <35), one for calf 2140 on 1 DPI and the other for calf 2417 on 3 DPI.

For calves in Group Two, nasal swabs were negative in 3/4 calves at all time points analyzed. In the remaining calf (2950), Ct values were in the suspect range on 1–2 DPI (Ct = 35.9 and 36.3, respectively) and positive on 4–5 DPI (Ct = 33.9 and 33.6, respectively). Virus isolation was attempted on all nasal swabs, and no live virus was isolated from nasal swabs from any calves at any time point for Group Two. All other samples collected in Group Two calves (saliva, serum, and ocular and fecal swabs) were negative in 4/4 at all time points analyzed.

In the necropsy samples, viral RNA was detected within and outside the respiratory tract tissues of Group One calves (Source data). In Group One calves necropsied at 6 DPI, viral RNA was detected with a Ct value < 35 in multiple tissue types, including multiple lung lobes, the palatine tonsils, and all four stomach compartments (rumen, reticulum, omasum, and abomasum) of both calves. At 13 DPI, viral RNA was also detected at a Ct value < 35 in the lung lobes and reticulum in one of the two calves in Group One. In Group Two, viral RNA was detected only in Calf 2950 necropsied at 6 DPI. Viral RNA was detected outside the respiratory tract of Calf 2950 in the retropharyngeal and tracheobronchial lymph nodes.

**Fig. 3:**
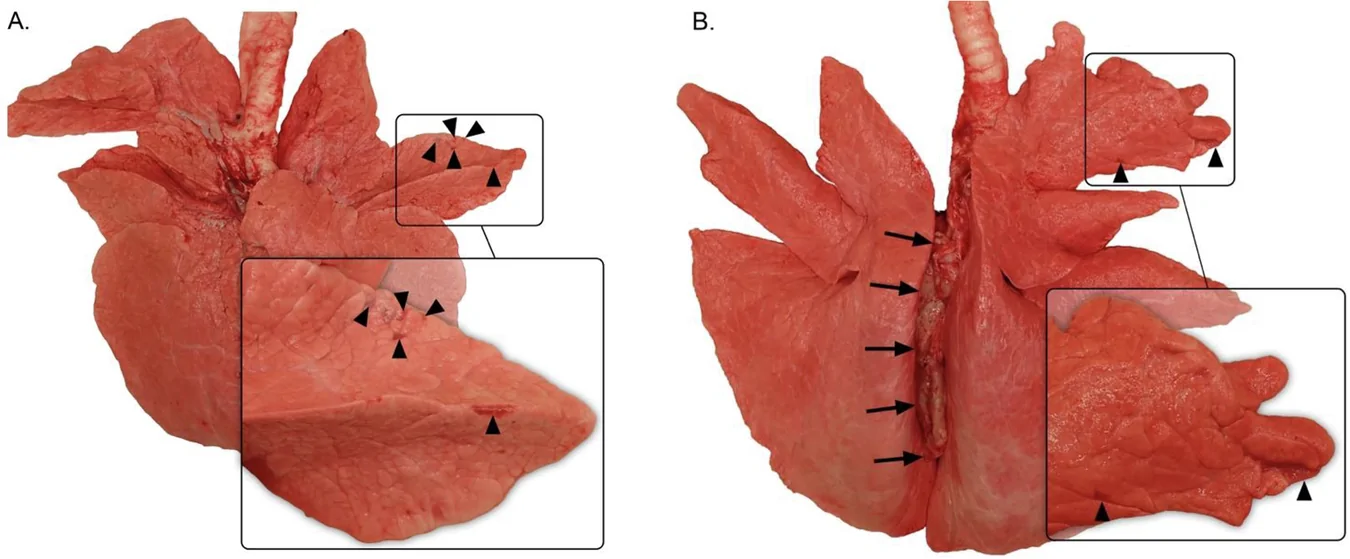
Lung lesions and lymphadenomegaly of Group One calves fed milk containing the virus. Minimal multifocal obstructive atelectasis (arrowheads) consistent with influenza A virus at 6 DPI in calf 2410 (**A**) and 2414 (**B**). A majority of the lung in both calves was macroscopically unremarkable and not denoted. **B** Lymphadenomegaly (arrows) of the mediastinal lymph node in calf 2414. Lung lesions and lymphadenomegaly were observed in 3 /4 calves in Group One. Lung lesions and lymphadenomegaly were not observed in the control calf. Source data are provided as a Source data file.

Histologic evaluation and IHC results by calf and tissue are summarized in the Source data. Two calves (2410 and 2414) from Group One were necropsied at 6 DPI. Minimal multifocal obstructive atelectasis was present in multiple lung lobes in both calves necropsied at 6 DPI (2410 and 2414) and in one of the calves necropsied at 13 DPI (2411; Fig. 3A, B; Supplementary Fig. 1; Source data). Gross pulmonary lesions were not observed in the control calf. Lymphadenomegaly was present in the medial retropharyngeal and mandibular lymph nodes of both calves, as well as in the parotid and mesenteric lymph nodes in calf 2410 and the mediastinal lymph node of calf 2414 at 6 DPI (Fig. 3B). Within the mesentery of 2410, adjacent to the mesenteric lymph node, there was a focal area of hemorrhage. Macroscopic evaluation of the remaining tissues was unremarkable.

**Fig. 4:**
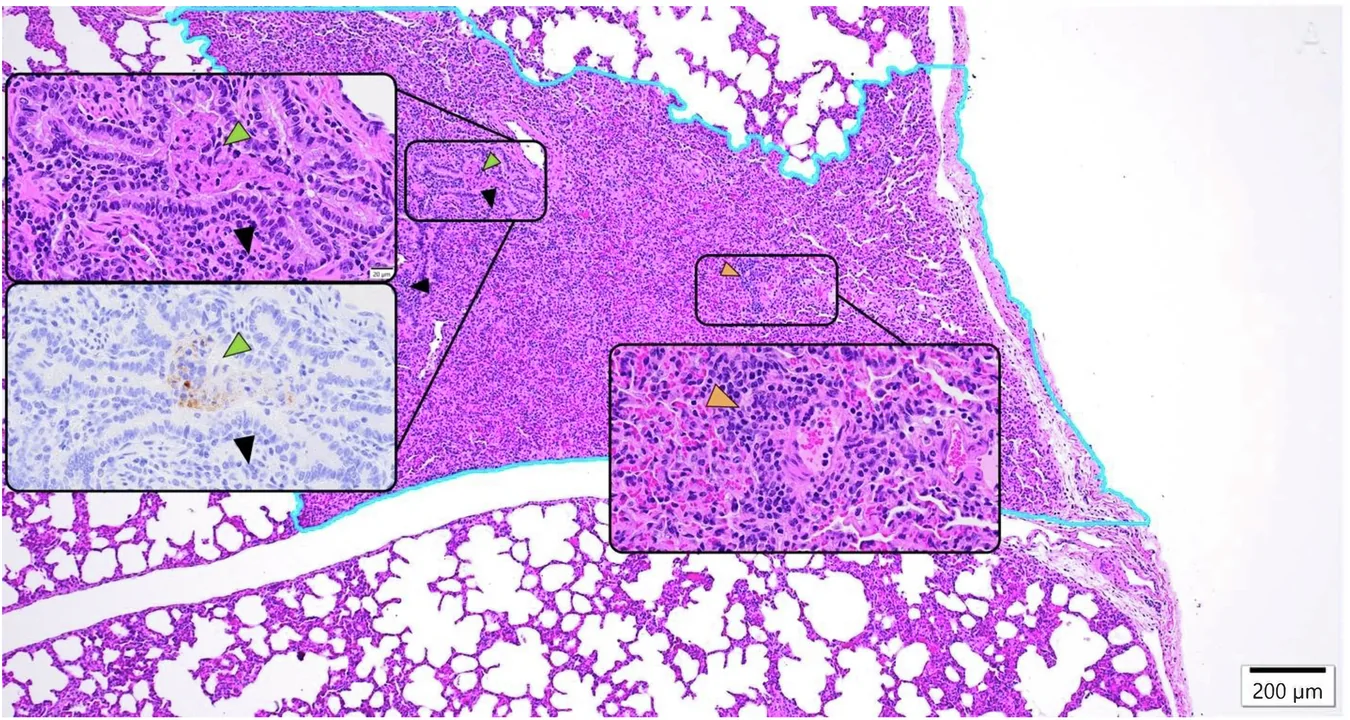
Bronchiolitis and pulmonary atelectasis in Group One Calf 2414. Accumulation of cellular debris (green arrowhead) in a bronchiole, peribronchiolar (black arrowhead) and perivascular (orange arrowhead) lymphocytic infiltrates, and influenza A virus nucleoprotein antigen detection (brown) by immunohistochemistry (bottom inset) in the luminal debris of the affected airway. Area of obstructive atelectasis outlined in blue. Bronchiolitis and pulmonary atelectasis were observed in 3 /4 calves in Group One. Bronchiolitis and pulmonary atelectasis were not observed in the control calf. Source data are provided as a Source data file.

**Fig. 5:**
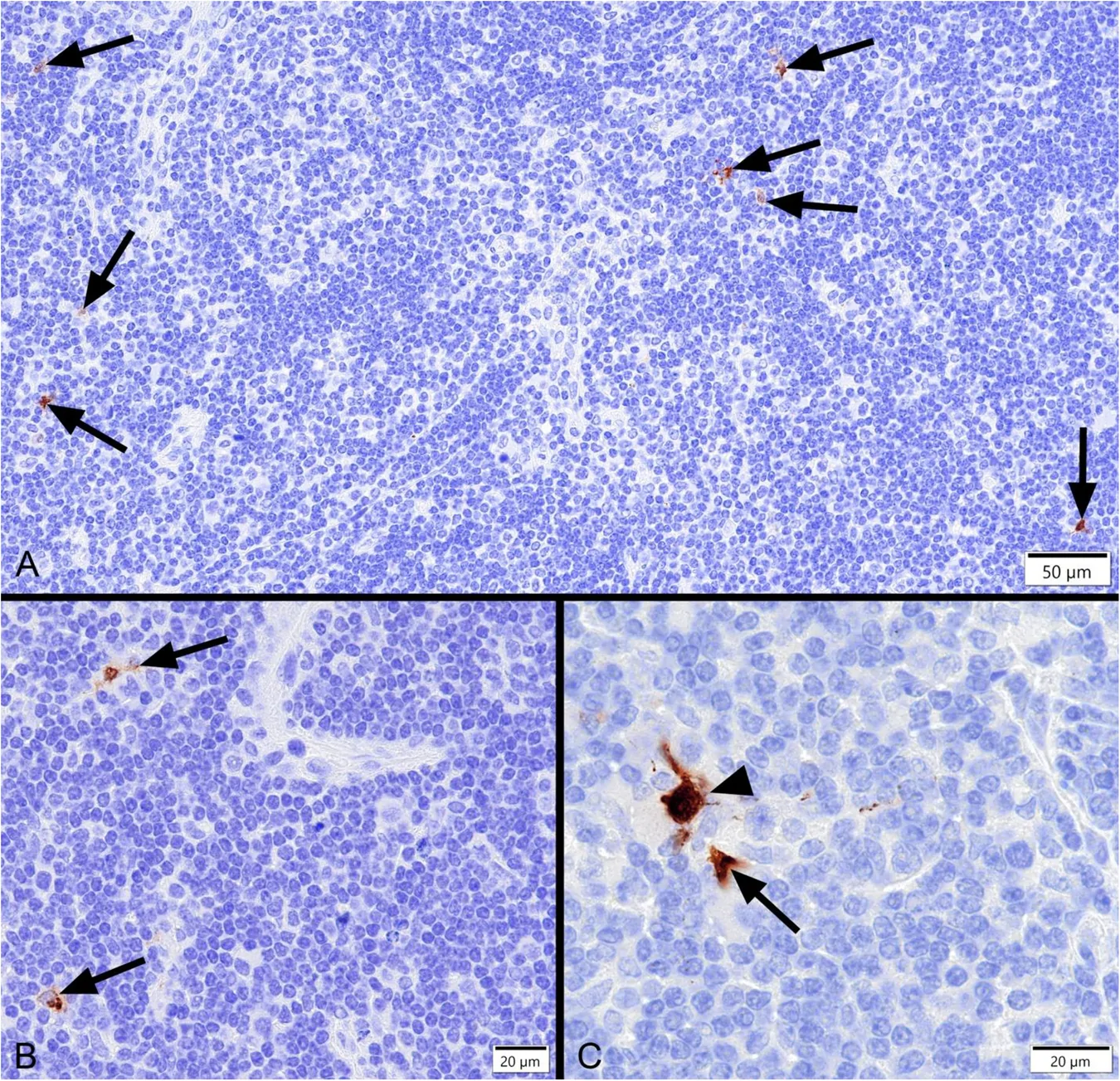
Influenza A virus antigen detection in Group One calf 2410. **A** Influenza A virus nucleoprotein antigen detection (brown) by immunohistochemistry in the cytoplasm (arrows) and nucleus (arrowhead) of leukocytes in the parotid lymph node (200×), **B** mandibular lymph node (400×), and **C** tracheobronchial lymph node (600×) of calf 2410 necropsied on DPI 6. Influenza A virus antigen was detected in the parotid lymph node of 2/4 calves, the mandibular lymph node of 2/4 calves, and the tracheobronchial lymph node of 2/4 calves. Influenza A virus antigen was not detected in the control calf. Source data are provided as a Source data file.

**Fig. 6:**
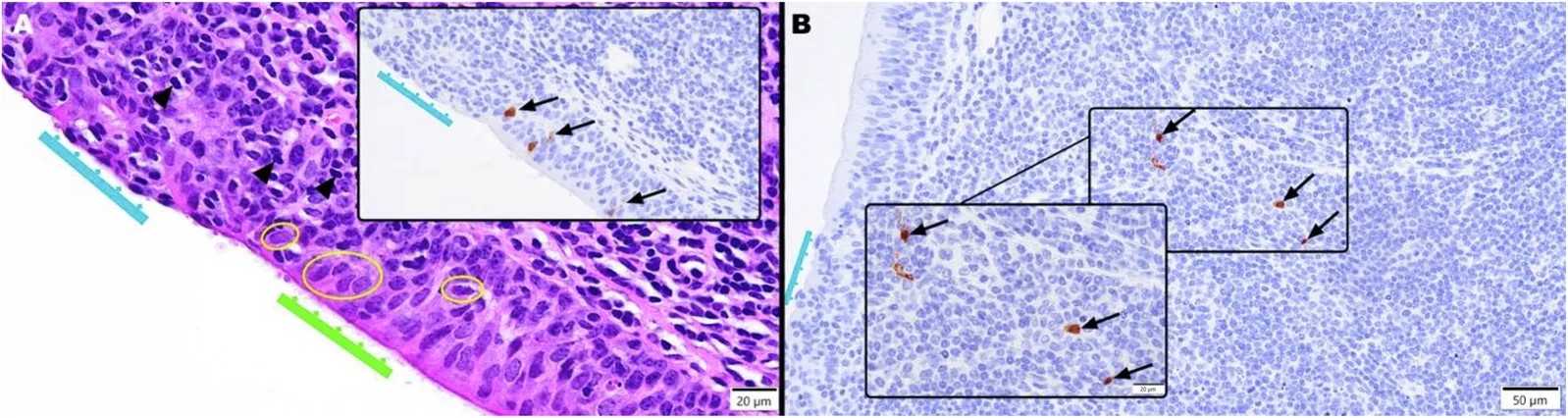
Influenza A virus detection in the pharyngeal tonsil of Group One Calf 2414. **A** Reticular epithelium (blue bar with arrows) with accumulation of intraepithelial lymphocytes (black arrowhead) adjacent to possible intranuclear viral inclusions (yellow circles) in the area near influenza A virus nucleoprotein antigen detection (arrows) by immunohistochemistry (inset). Intact cilia are denoted by the green bar with arrows. **B** Reticular epithelium (blue bar with arrows) with subjacent detection of influenza A virus nucleoprotein antigen (arrows) by immunohistochemistry (inset). Influenza A virus detected in the pharyngeal tonsil of ¼ calves, Group One. Influenza A virus was not detected in the pharyngeal tonsil of the control calf. Source data are provided as a Source data file.

Minimal to mild histologic lesions were noted in the lung sections from Calf 2410 and 2414. Lesions included bronchiolitis obliterans, luminal cellular debris in bronchioles with adjacent atelectasis, and perivascular lymphocytic infiltrates (Fig. 4). IAV antigen was detected by IHC in the luminal debris of a single bronchiole in the middle lung lobe of Calf 2414 (Fig. 4). Mild to moderate parafollicular hyperplasia was observed in multiple lymph nodes (Supplementary Data Fig. 2). IAV antigen was detected by IHC in the cytoplasm and nucleus of leukocytes in the retropharyngeal lymph node, mandibular lymph node, parotid lymph node, and tracheobronchial lymph node of both calves necropsied on DPI 6 (Fig. 5; Supplementary Fig. 3). IAV antigen was detected by IHC in the pharyngeal tonsil (epithelial layer and subjacent leukocytes) and palatine tonsil (leukocytes) of Calf 2414 (Fig. 6A, B). Mild, multifocal erosion with loss of goblet cells of the conjunctiva was also observed from Calf 2410 that had an ocular swab with positive RT-qPCR, but IAV was not detected by IHC. Microscopic evaluation of the negative control calf was unremarkable, and IAV antigen was not detected by IHC (Source data).

Two calves (2950 and 2958) from Group Two were necropsied at 6 DPI. Minimal multifocal obstructive atelectasis was present in multiple lung lobes in one calf (2958) necropsied at 6 DPI and in one of the calves necropsied at 13 DPI (2957). Lymphadenomegaly was not observed, and macroscopic evaluation of the remaining tissues was unremarkable. Influenza A virus nucleoprotein antigen was detected by IHC in one tissue (retropharyngeal lymph node) in one calf (2950) (Supplementary Fig. 4)

### Serological responses

All calves were negative for NP ELISA antibody in serum prior to inoculation and at 6 DPI for both groups of calves. The two remaining calves from Group One were positive on 10 DPI using a sample/negative (S/N) ratio cut-off of ≤0.6. The sham inoculated negative control remained seronegative as expected (data not shown). At 13 DPI, the calves from Group One continued to be positive for NP antibody and became positive for H5 ELISA antibody and HI antibody, as shown in Table 2. The two remaining calves from Group Two were negative at both 10 DPI and 13 DPI.

**Table 2:**
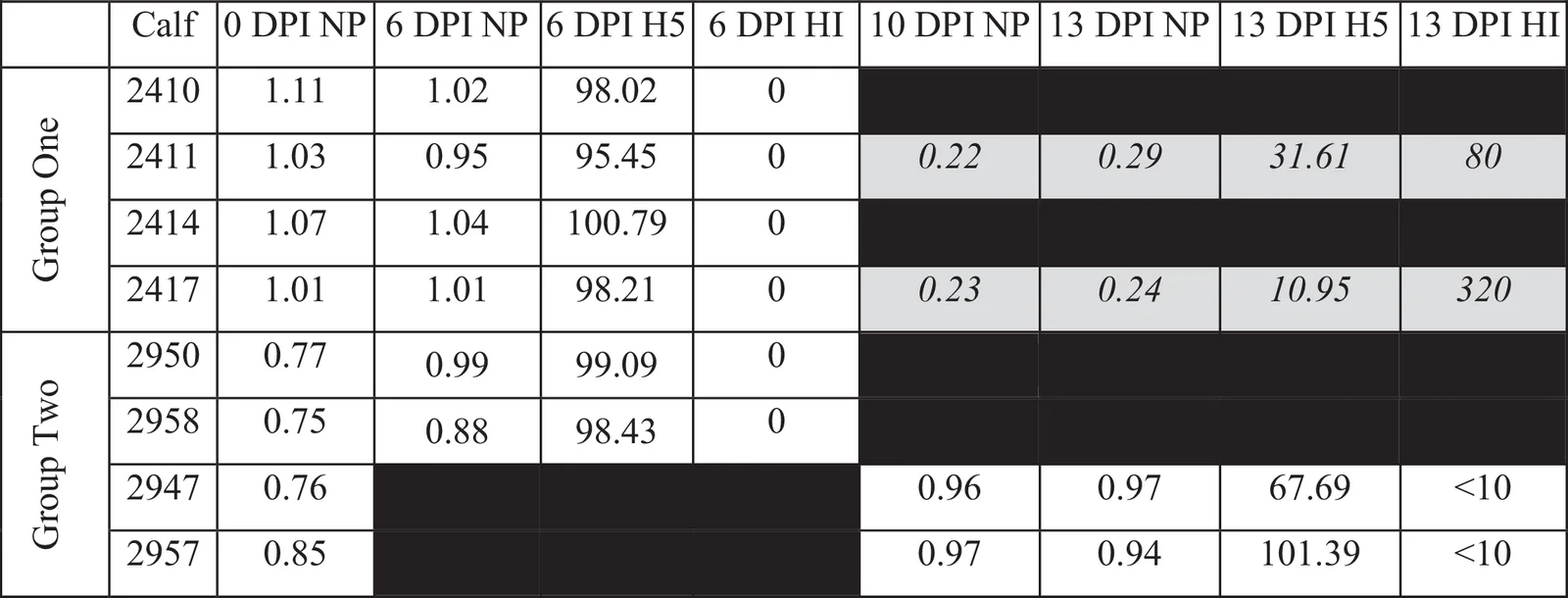
Serum antibody detection in calves fed milk with virus (Group One) and milk with virus and antibody (Group Two)

NP ELISA sample to negative(S/N) ratio of optical densities, H5 ELISA sample to negative(S/N) percentage, and hemagglutinin inhibition (HI) reciprocal titer. Positive sample indicated by gray shading and italics; black indicates not tested. Four animals (two from each group) were euthanized on 6 DPI.

## Discussion

This manuscript describes the first model of experimental infection of calves fed raw milk from cows inoculated with the H5N1 genotype B3.13 strain. Seroconversion confirms exposure from this route of transmission. Additionally, calves developed clinical signs including nasal discharge, mild fever, mild lethargy, loose stool, and slightly increased respiratory effort for 5–6 days, had RT-qPCR and virus isolation positive samples, and lesions consistent with an active influenza A virus infection. While the viral load in the raw milk fed to calves was high, as shown in Table 3, viral replication in calves was at a low level with many RT-qPCR values close to the limit of detection. Nevertheless, a viable virus was isolated from nasal swabs and tissue samples, as shown in Fig. 2 and Supplementary Table 2. Live virus was isolated from nasal swabs from calves from 2 to 5 DPI, with continued detection after calves were transitioned to being fed milk from non-inoculated cows. Ct values of the nasal swabs were significantly correlated (−0.55) with lethargy in the calves. As Ct values dropped and viral load increased, calves became more lethargic. Still, signs of clinical disease were mild and may not be recognized or attributed to HPAI under field conditions with other environmental or health stressors. The clinical signs observed in calves, including nasal discharge and loose stool, are common in pre-weaned dairy calves and could easily be attributed to other etiologies under field conditions. Digestive and respiratory pathogens in dairy calves continue to be a major problem for dairy producers^13^ and account for over 80% of morbidity events in pre-weaned dairy calves (50.9% digestive, 28.1% respiratory, 5.4% digestive and respiratory)^14^. Furthermore, calves are often transported off farm, with 1 of every 10 operations in the U.S. raising dairy heifers off-site, and most veal and dairy-beef calves are transported to another facility for raising^15^. Therefore, infected calves with mild clinical signs could be a source of farm-to-farm transmission and warrant future studies investigating calf-to-calf transmission and the adaptation of H5N1 to the bovine respiratory tract.

**Table 3:** Influenza A virus detection in the milk fed to calves containing virus (Group One) and virus and antibody (Group Two) reported as Ct values and virus titer equivalents

Group 1 milk without antibodies			Group 2 milk with antibodies		
Day	Ct value	Titer equivalent	Day	Ct value	Titer equivalent
0	37.0	3.04E+02	0	21.14	1.11E+08
1	18.5	9.38E+08	1	26.7	1.25E+06
2	24.7	6.28E+06	2	21.1	1.15E+08
3	29.0	1.95E+05	3	28.1	4.03E+05

Macroscopic and/or microscopic lesions were observed in the lung, lymph nodes, and conjunctiva. IAV antigen was detected by IHC in the lymph nodes of the head and respiratory tract (Calf 2410 and 2414), tonsils (Calf 2414), and one lung section in the luminal debris of a single conducting airway affected by bronchiolitis (Calf 2414). The presence of IAV antigen in the nuclei of leukocytes in lymph nodes is consistent with IAV replication in these cells and suggests the possibility of immune trafficking of infected cells that could infect other cells in other tissues.^16,17^ Detection of IAV antigen in the epithelial layer of the pharyngeal tonsil suggests this may be an additional site of IAV replication in cattle and requires further investigation. The presence of erosive conjunctivitis in Calf 2410 aligns with the positive RT-qPCR ocular swab at 3 DPI; however, IAV was not detected by IHC. This may be a result of the time between detection by RT-qPCR and necropsy at 6 DPI. Conjunctivitis was not observed macroscopically.

Other work has shown mild respiratory disease and lung lesions with concurrent detection of IAV by IHC in yearling heifers experimentally inoculated with HPAI through an aerosol respiratory route^9^. However, the calves in the study reported here had consistent nasal swab detection over subsequent days and lower Ct values compared to aerosol-inoculated heifers. Furthermore, IAV was detected by IHC in additional tissue types. These data, along with seroconversion of the Group One calves necropsied at 13 DPI, confirm infection from this route of inoculation. The minimal detection of IAV antigen within positive tissue sections in both studies may be a result of necropsy timing and the minimal macroscopic lesions for which to select histologic sections. Evaluating tissues at peak infection around 3 DPI may provide additional insights into viral pathogenesis and tissue and/or cellular tropism.

This work also demonstrates that the presence of neutralizing antibodies in the milk can impact H5N1 transmission, as antibodies prevented the transmission of H5N1 genotype B3.13 to calves. No clinical signs of disease nor seroconversion were observed in these calves (Group Two). In 3/4 calves, we did not detect any viral RNA or antigen in any samples collected ante- or post-mortem. However, viral RNA was detected in nasal swabs from one of the four calves on DPI 4 and DPI 5, and IAV antigen was detected by IHC in the retropharyngeal lymph node of the same calf on 6 DPI. The lack of seroconversion supports the observation that transmission was prevented in the 2 calves that survived to 13 DPI, but additional time points are warranted in future studies.

Milk diverted from the human food supply in H5N1-positive dairy herds or from suspect cows should not be fed to calves without pasteurization. The continued transmission of HPAI in dairy cattle is an animal health crisis due to the associated morbidity, mortality, interspecies transmission events, and economic losses, and is a public health challenge due to occupational exposure on dairy farms. The determination of routes of transmission and of mechanisms of protection are essential steps to inform subsequent research for the development of successful intervention strategies.

## Methods

### Animal inoculations

All animal work was carried out in a BSL-3-Ag facility in compliance with a protocol approved by the Institutional Animal Care and Use Committee of the USDA Agricultural Research Service National Animal Disease Center (NADC). Four adult Holstein lactating cattle free of influenza A virus and antibody were obtained from Iowa State University and moved into BSL-3 containment as part of a larger study described elsewhere. Following an acclimation period, cows were inoculated via the intramammary route as described in Baker et al.^9^ with the modification of 1 ml of 1 × 10^2^ (right front quarter), 10^4^ (left front quarter), or 10^6^ (left rear quarter) TCID_50_/ml A/dairy cattle/Texas/24-008749-002/2024. Milk from these cows was used to inoculate calves as described below.

Eight Holstein calves (five females and three males), approximately 7–11 weeks old, born on the NADC campus, were moved into BSL-3 containment and allowed to acclimate for 2 days. One additional calf (male) remained in routine production conditions as a negative control and was fed milk from healthy cows. Calves were fed 0.95 L of unpasteurized milk twice a day (AM and PM) via bucket throughout the studies. In Group One, 4 calves were given milk pooled from two cows during 1 through 4 days post-inoculation (DPI). In Group Two, 4 calves were given 0.95 L of milk from two in mid-infection following seroconversion (15–19 DPI), spiked with 187.5 ml of milk from a cow early in infection (3 DPI) to keep viral load as similar as possible to Group One. After day four of each study, all calves were fed raw milk obtained from non-inoculated cows housed at NADC’s own dairy herd. The negative control calf was fed non-inoculated milk throughout the course of the study.

The presence of virus RNA from a pool of each day’s milk fed from inoculated cows in Groups One and Two was confirmed by an influenza A virus RT-qPCR, as described below. The presence of antibodies in the milk fed to calves in Group Two was confirmed individually in each inoculated cow, as described below.

### Clinical evaluation and sample collection

Behavior (lethargy, response to stimulus such as personnel entering the pen, eating, and drinking), respiratory effort, rectal temperatures, and clinical samples were monitored and/or collected daily from calves for 6 days post-inoculation (DPI). Criteria for clinical scores used to assess dairy calves are shown in Supplementary Table 1. Ocular, nasal, and rectal samples were collected with FLOQ nylon swabs (Copan, Murrieta, CA) and placed into molecular transport media (PrimeStore MTM, Longhorn, Bethesda, MD). Blood was collected via jugular venipuncture and transferred into serum separator tubes. Tubes were centrifuged, and serum was aliquoted. Saliva was collected with an absorbent pad (Super-SAL, Oasis Diagnostics, Vancouver, WA). All samples were taken prior to feeding milk each day.

### Virus detection in clinical samples

Milk pooled for feeding, swabs (ocular, nasal, and rectal), serum, saliva, and tissue samples were tested using an influenza A virus (IAV) RT-qPCR kit, as previously described^18^. Briefly, viral RNA was extracted from milk, swabs, serum, and saliva using the MagMAX™−96 Viral RNA Isolation Kit (Thermofisher Scientific, catalog #AMB18365) following the manufacturer’s instructions. RNA extraction from tissues was processed through the spin procedure of the MagMAX™−96 for Microarrays Total RNA Isolation Kit (Thermofisher Scientific, catalog #AM1839). The extracted product was subjected to RT-qPCR using the VetMAX-Gold SIV Detection kit (Life Technologies, catalog # 4415200). Ct values less than 35 were considered positive, while Ct values greater than 38 were considered negative. Ct values 35–38 were considered suspect. All nasal swabs and tissues with RT-qPCR Ct <35 were inoculated onto 10-day-old embryonating chicken eggs to determine the presence of viable virus.

### Antibody detection

Seroconversion was determined using a blocking ELISA to detect antibodies to the nucleoprotein (NP) (Influenza A Ab, IDEXX, Westbrook, Maine), according to the manufacturer’s instructions, with a 1:5 starting dilution for serum. The cut-off sample to negative (S/N) optical density (O.D.) ratio was ≤ 0.6. Serum samples were also tested using a competition multi-species ELISA to detect antibodies against the hemagglutinin H5 (ID Screen® Influenza H5 Antibody Competition 3.0 Multi-Species, Innovative Diagnostics, Grabels, France), according to the manufacturer’s instructions. Samples were positive when the percentage of the test sample to the kit negative control ratio (S/N) was ≤0.4.

H5-specific hemagglutination inhibition (HI) was conducted as described previously^19,20^, with an H5 clade 2.3.4.4b hemagglutinin gene from A/Bald eagle/FL/W221340P/2022 engineered to be low pathogenicity (kindly provided by Dr. Richard Webby, St. Jude’s Children’s Research Hospital) for the calf sera and the homologous challenge strain for the cow sera. Prior to HI, serum samples were heat-inactivated at 56 °C for 30 min, treated with receptor-destroying enzyme (Hardy Diagnostics, Santa Maria, CA), and adsorbed with 100% rooster red blood cells for 60 min to remove nonspecific hemagglutinin inhibitors and natural serum agglutinins. A reciprocal HI titer ≥40 was considered positive. Virus neutralization assays with milk from inoculated cows for the Group Two feeding were conducted on the London line of Madin-Darby Canine Kidney (MDCK) cells, as described previously in Baker et al.^9^.

### Macroscopic and microscopic evaluation

All cattle were humanely euthanized by a veterinarian via sedation with a mixture of xylazine (Drecha, Overland Park, KS) and ketamine (VetOne, Boise, ID) intramuscularly, followed by intravenous administration of pentobarbital sodium (Fatal Plus, Vortech Pharmaceuticals, Dearborn, MI). Two inoculated calves from each milk feeding group and the negative control calf were necropsied at 6 DPI, while the remaining two inoculated calves from each group were necropsied at 13 DPI. At the time of necropsy, the thoracic cavity, abdominal cavity, nasal cavity, and cranium (longitudinal section) underwent macroscopic evaluation. Paired fresh and formalin-fixed tissues for RT-qPCR and microscopic evaluation, respectively, were collected (Supplementary Tables 2 and 3). Formalin-fixed tissues were processed routinely for microscopic evaluation. Additional fresh samples included tracheal swab, urine, feces and rumen, omasum, abomasum, and reticulum contents.

Immunohistochemistry (IHC) targeting the nucleoprotein (NP) antigen of Influenza A virus (IAV) was performed as previously described, with the inclusion of a positive and negative tissue control^16^. A representative section was selected for IHC from the ethmoids, nasal turbinates, and trachea. IHC was performed on all sections of the lung, tonsil, and lymph nodes. Additional tissue sections with RT-qPCR detection and/or histologic lesions were also evaluated by IHC for the detection of NP antigen to confirm a causal link between IAV and the lesion(s).

### Statistical analysis

Pearson correlation coefficients between Ct values for nasal swabs and clinical observations for all time points were calculated using the package Hmisc v 5.-3c in RStudio 2002.02.1 = 461 “Prairie Trillium.” Correlations were considered significant at *P* < 0.05.

### Reporting summary

Further information on research design is available in the Nature Portfolio Reporting Summary linked to this article.

## Supplementary information

Supplementary Information

Reporting Summary

Transparent Peer Review file

## Data Availability

Datasets generated and/or analyzed during the current study are appended as supplementary data. Source data are provided with this paper and are available at https://doi.org/10.6084/m9.figshare.3299641, and macroscopic and microscopic tissue findings are available at https://doi.org/10.6084/m9.figshare.29066585.
