# Supplementary material for: Susceptibility of calves fed unpasteurized milk from cows infected with highly pathogenic avian influenza H5N1
Source: Nat Commun. 2026 Aug 21;17:10036. doi: 10.1038/s41467-026-76300-1 (PMC13594346; doi:10.1038/s41467-026-76300-1)
Supplement: Supplementary file 1 [file 41467_2026_76300_MOESM1_ESM.pdf]

Supplementary Figures and Tables

Supplementary Table 1. Clinical scores used to assess dairy calves.

Score	*Behavior*	*Respiratory signs/Rate*	*Cough*	*Nasal discharge*	*Ocular Discharge*	*Feces*
0	Normal	Normal	Normal	Normal, serous discharge	Normal	Normal
1	Mild lethargy with decrease in ambulation and attitude compared to pen mates	Slightly increased respiratory effort/slight dyspnea	Induce single cough	Small amount of unilateral, cloudy discharge	Mild ocular discharge (predominately in canthi)	Semi-formed, pasty
2	Moderate lethargy with stimulation needed to provoke ambulation	Notable increase in respiratory effort/dyspnea	Induce repeated coughs or occasional spontaneous cough	Cloudy or excessive mucus	Moderate bilateral ocular discharge	Loose, but stays on top of bedding or dry and tacky
3	Marked lethargy with ambulation not provoked by stimulation	Severe dyspnea with respiratory distress and tachypnea	Repeated spontaneous coughing	Copious, mucopurulent nasal discharge	Heavy ocular discharge (lashes coated) swelling/erythema of the eyelid	Watery, sifts through bedding

*Adapted from https://www.vetmed.wisc.edu/fapm/svm-dairy-apps/calf-health-scorer-chs/ Calf Health Scorer (CHS) – Food Animal Production Medicine – UW–Madison (wisc.edu)

Supplementary Table 2. RT-qPCR results of tissues and samples collected at necropsy from Group One calves fed milk with virus but not antibody. Virus isolation was attempted for all tissues with Ct< 38. Red fill denotes live virus was successfully isolated from the sample

Tissue	DPI 6		DPI 13	
	**2410**	**2414**	**2411**	**2417**
Tracheal swab	40.0	34.3	40.0	40.0
Olfactory bulb	34.69	40.0	40.0	40.0
Cerebrum	38.95	40.0	40.0	40.0
Cerebellum	40.0	40.0	40.0	40.0
Brainstem	40.0	40.0	40.0	40.0
Pharyngeal tonsil	40.0	35.08	40.0	40.0
Palatine tonsil	30.91	33.78	40.0	40.0
Lingual tonsil	40.0	40.0	40.0	36.96
Cranial part right cranial lung lobe	40.0	40.0	40.0	40.0
Caudal part right cranial lung lobe	40.0	40.0	40.0	40.0
Cranial part left cranial lung lobe	40.0	40.0	40.0	40.0
Caudal part left cranial lung lobe	40.0	40.0	40.0	40.0
Middle lung lobe	40.0	40.0	40.0	40.0
Accessory lung lobe	32.27	33.78	40.0	40.0
Caudal right lung lobe	33.97	37.39	40.0	40.0
Caudal left lung lobe	40.0	40.0	40.0	40.0
Retropharyngeal lymph node	37.62	29.66	40.0	40.0
Mandibular lymph node	31.94	32.11	40.0	40.0
Parotid lymph node	33.93	40.0	40.0	40.0
Tracheobronchial lymph node	37.17	33.63	40.0	40.0
Supramammary lymph node	40.0	40.0	40.0	40.0
Inguinal lymph node	40.0	40.0	40.0	40.0
Mesenteric lymph node	40.0	40.0	40.0	40.0

Heart	40.0	40.0	40.0	40.0
Thymus	40.0	40.0	40.0	40.0
Spleen	40.0	40.0	40.0	40.0
Pancreas	40.0	40.0	40.0	40.0
Liver	40.0	40.0	40.0	40.0
Kidney	40.0	40.0	40.0	40.0
Rumen	33.89	34.76	35.43	37.61
Reticulum	33.45	33.81	34.71	38.72
Omasum	34.24	34.58	35.80	40.00
Abomasum	33.51	33.80	36.82	40.0
Jejunum	40.0	40.0	40.0	40.0
Spiral colon	40.0	39.47	40.0	40.0
Descending colon	40.0	40.0	40.0	40.0
Urinare	40.0	40.0	40.0	40.0
Diaphragm	40.0	40.0	40.0	
Pectoral muscle (Brisket)	40.0	40.0	40.0	40.0

Each column represents an individual animal. Red indicates samples with positive virus isolation and black a sample that was not collected.

Supplementary Table 3. Tissues and samples collected at necropsy and tested by RT-qPCR for Group Two calves fed milk from inoculated cows shedding virus and neutralizing antibodies.

Tissue	DPI 6		DPI 13	
	2950	2958	2947	2957
Olfactory bulb	40.0	40.0	40.0	40.0
Cerebrum	40.0	40.0	40.0	40.0
Pharyngeal Tonsil	36.27	40.0	40.0	40.0
Palatine Tonsil	40.0	40.0	40.0	40.0
Lingual Tonsil	40.0	40.0	40.0	40.0
Cranial part right cranial lung lobe	40.0	40.0	40.0	40.0
Caudal part right cranial lung lobe	40.0	40.0	40.0	40.0
Cranial part left cranial lung lobe	40.0	40.0	40.0	40.0
Caudal part left cranial lung lobe	40.0	40.0	40.0	40.0
Middle lung lobe	40.0	40.0	40.0	40.0
Accessory lung lobe	40.0	40.0	40.0	40.0
Caudal right lung lobe	40.0	40.0	40.0	40.0
Caudal right lung lobe	40.0	40.0	40.0	40.0
Retropharyngeal lymph node	31.99	40.0	40.0	40.0
Mandibular lymph node	40.0	40.0	40.0	40.0
Parotid lymph node	40.0	40.0	40.0	40.0
Tracheobronchial lymph node	32.67	40.0	40.0	40.0
Rumen	40.0	40.0	40.0	40.0
Reticulum	40.0	40.0	40.0	40.0
Omasum	40.0	40.0	40.0	40.0
Abomasum	40.0	40.0	40.0	40.0

Each column represents an individual animal. Red indicates samples with positive virus isolation and black a sample that was not collected.

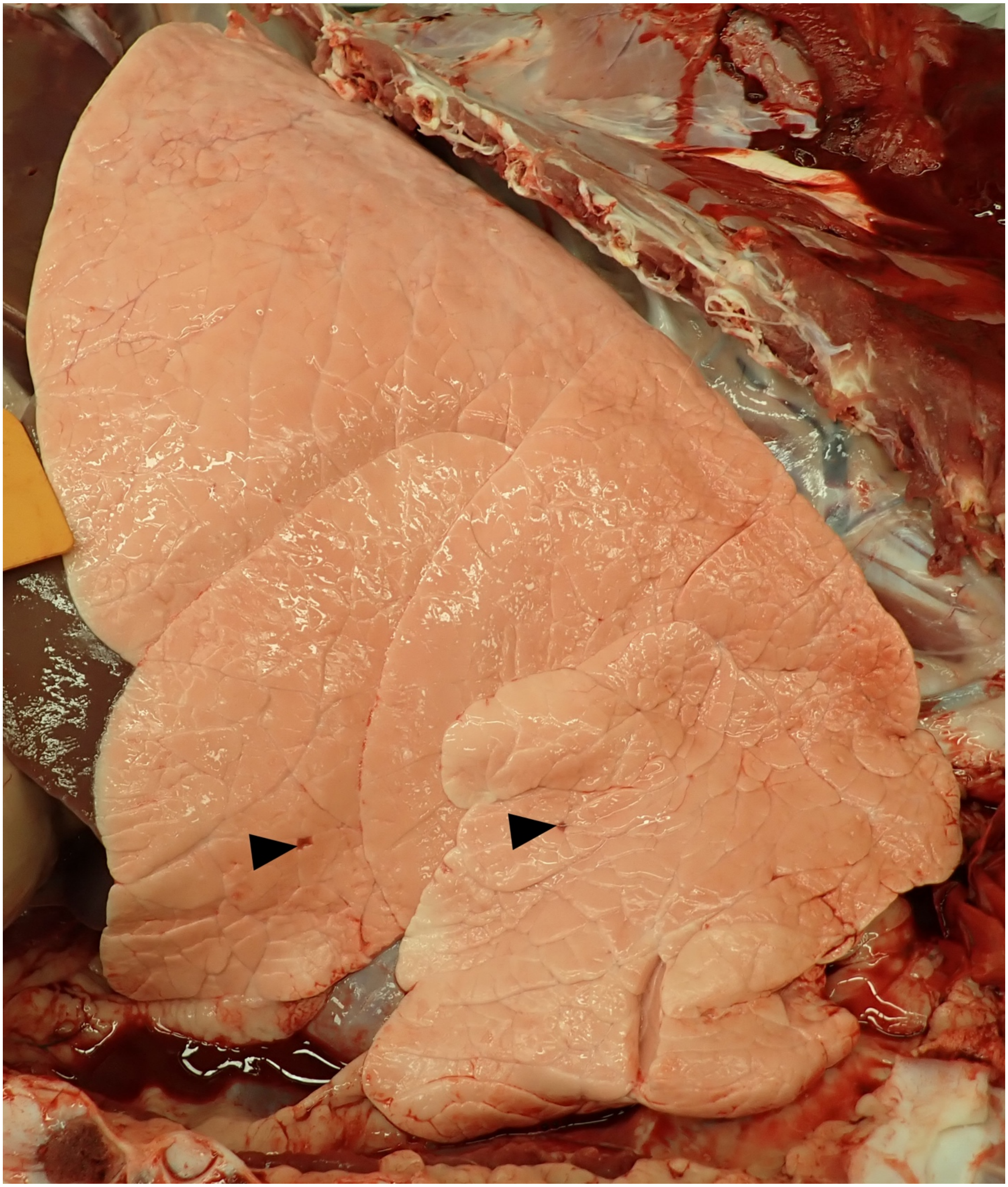

Supplementary Figure 1. Minimal multifocal obstructive atelectasis (arrowheads) consistent with influenza A virus at 6 dpi in calf 2411. A majority of the lung is macroscopically unremarkable and not denoted.

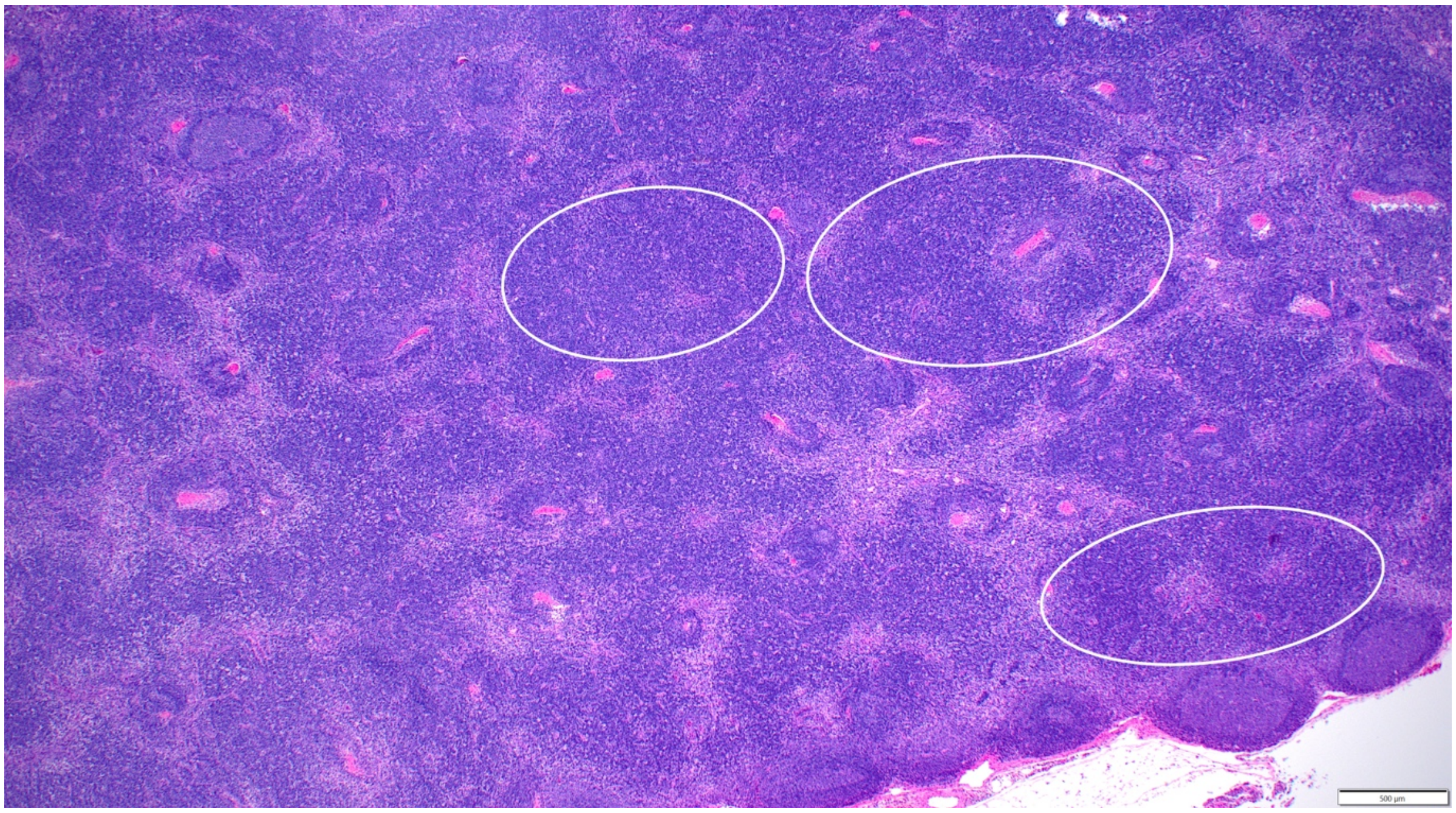

Supplementary Figure 2. Parafollicular hyperplasia (white circles) of the tracheobronchial lymph node in Calf 2414.

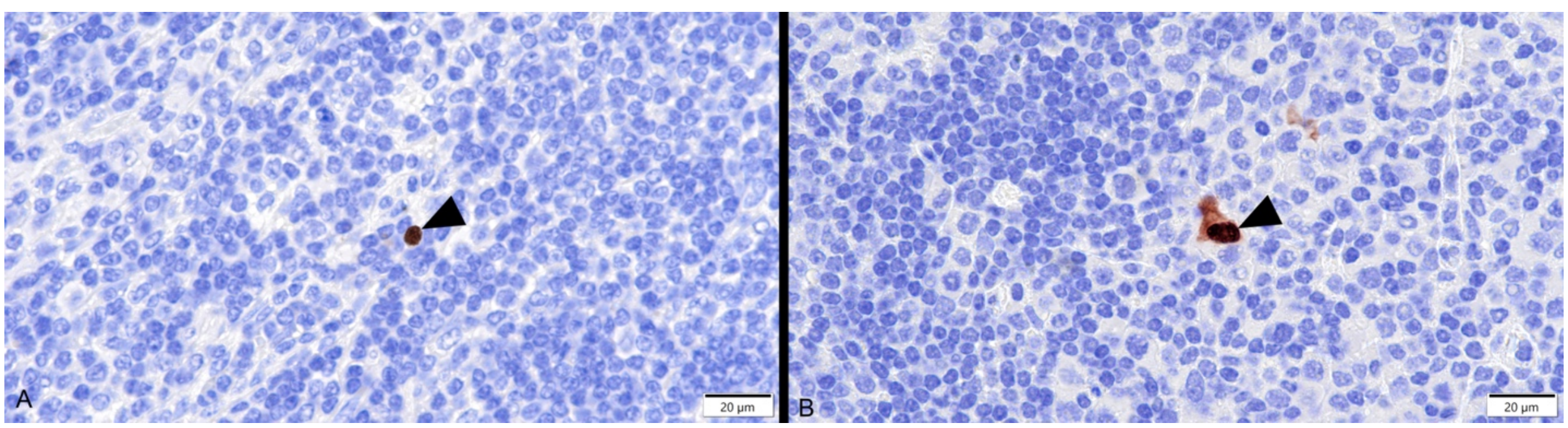

Supplementary Figure 3. Influenza A virus nucleoprotein antigen detection (brown) by immunohistochemistry in the nuclei (arrowhead) of leukocytes with different morphologies consistent with different cell types in the retropharyngeal lymph node of 2410 (A) and 2414 (B) necropsied on DPI 6.

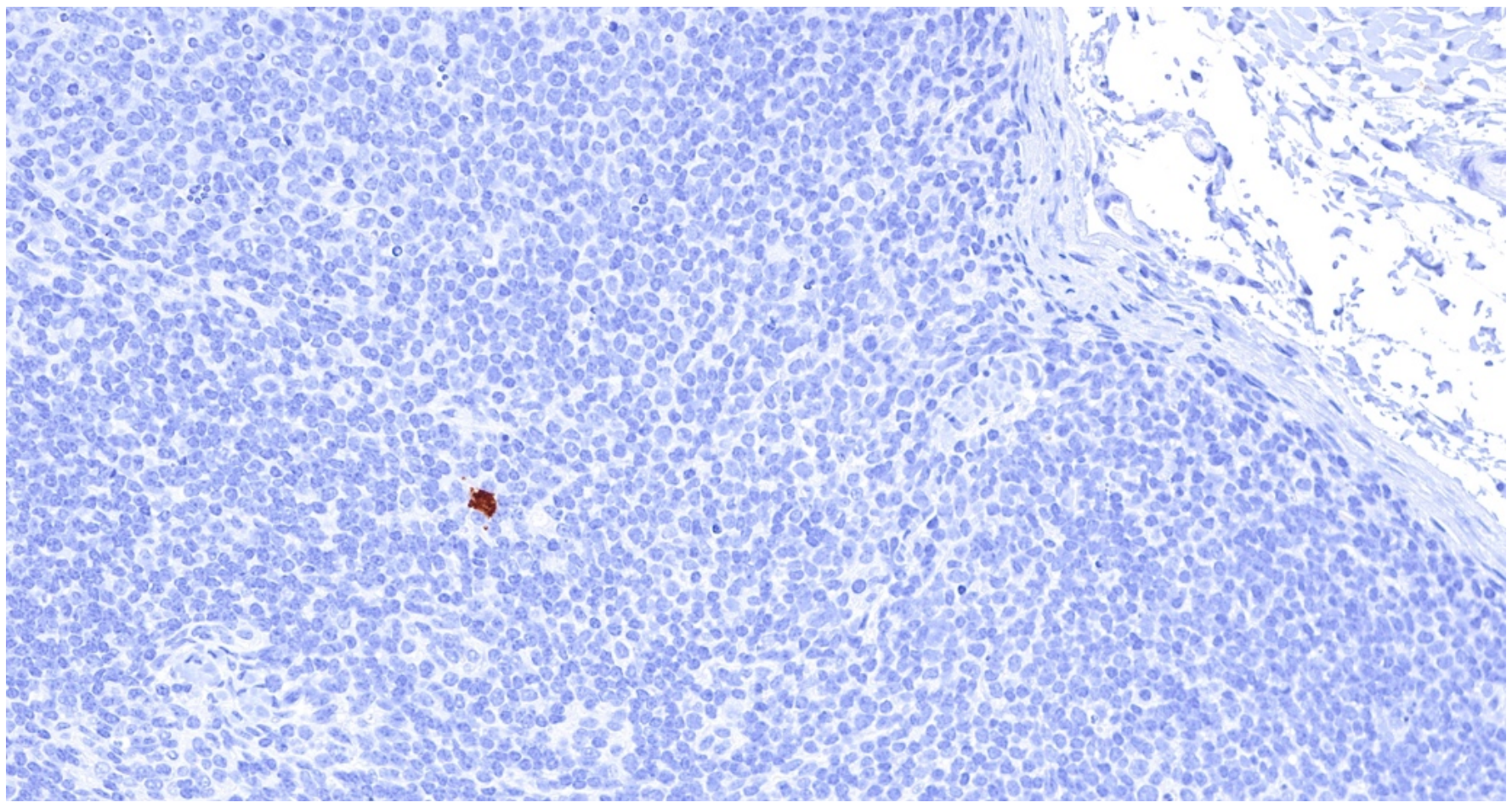

Supplementary Figure 4. Influenza A virus nucleoprotein antigen detection (brown) by immunohistochemistry in a leukocyte in the retropharyngeal lymph node of 2950 necropsied on DPI 6.

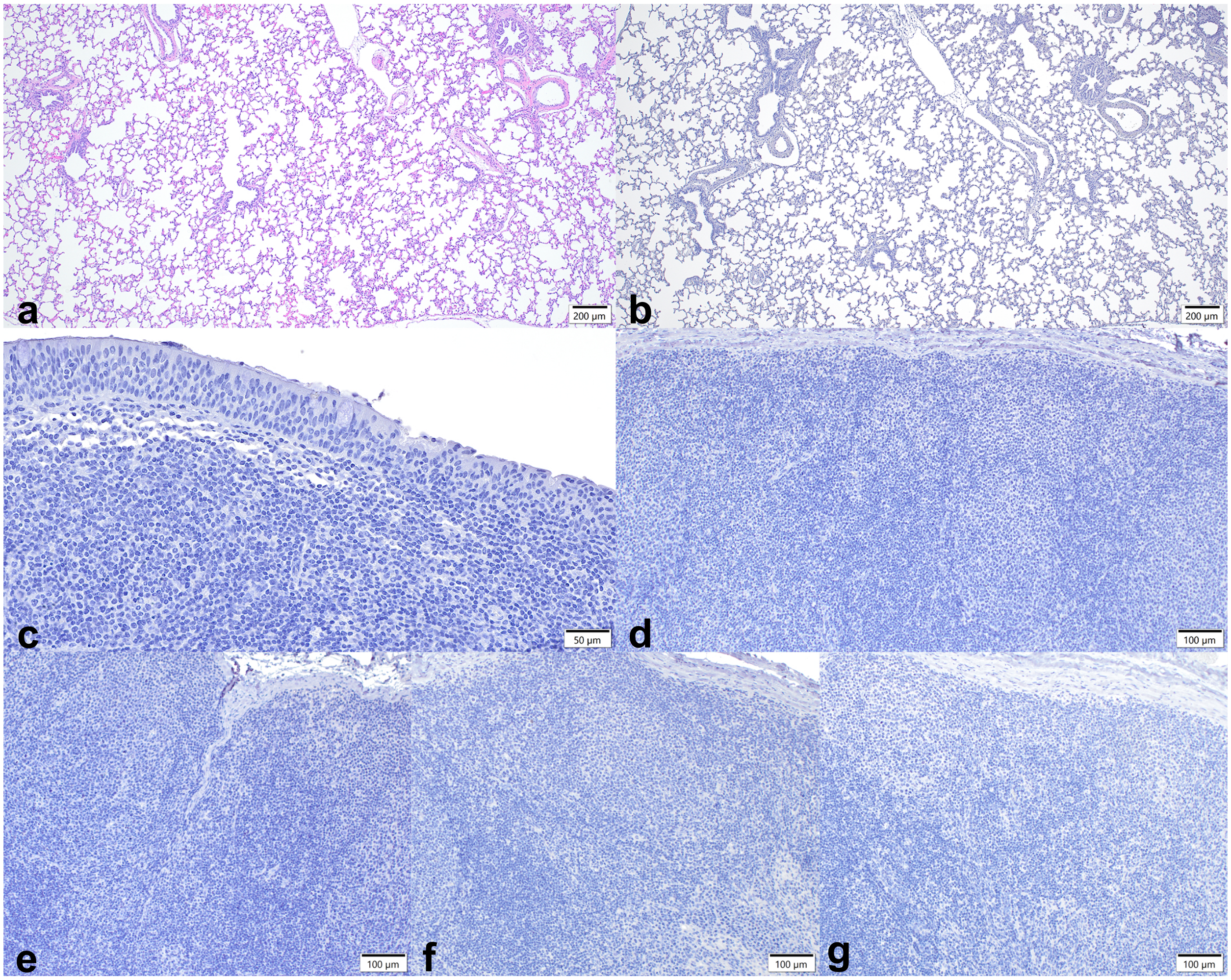

Supplementary Figure 5. Histologically unremarkable lung (A) and no antigen detection of Influenza A virus nucleoprotein by immunohistochemistry in the lung (B), pharyngeal tonsil (C), retropharyngeal lymph node (D), tracheobronchial lymph node (E), mandibular lymph node (F), and parotid lymph node (G) of Calf 2419 (negative control) necropsied on DPI 6.
