# Supplementary material for: Susceptibility of calves fed unpasteurized milk from cows infected with highly pathogenic avian influenza H5N1
Source: Nat Commun. 2026 Aug 21;17:10036. doi: 10.1038/s41467-026-76300-1 (PMC13594346; doi:10.1038/s41467-026-76300-1)
Supplement: Supplementary file 3 [file 41467_2026_76300_MOESM3_ESM.pdf]

nature portfolio

Peer Review File

Susceptibility of calves fed unpasteurized milk from cows experimentally infected with highly pathogenic avian influenza H5N1

Corresponding Author: Dr Kaitlyn Sarlo Davila

This file contains all reviewer reports in order by version, followed by all author rebuttals in order by version.

Version 0:

Reviewer comments:

Reviewer #1

(Remarks to the Author)
The study by Sarlo Davila et al. tested the susceptibility of calves to infection with highly pathogenic avian influenza H5N1via feeding with milk from infected cows. Given the continuing problems caused by highly pathogenic avian influenza H5N1 in dairy cattle in the US this is an important question to answer. Calves fed infected milk without antibodies showed clinical signs including nasal discharge, mild fever, loose stool and slightly increased respiratory effort for 5-6 days. Virus could be detected in multiple tissues and calves seroconverted. Calves fed infected milk that also contained maternal H5-specific antibodies did not develop clinical symptoms and only 1/4 calves had detectable virus and no seroconversion was detected.
The study sheds light on calves fed with unpasteurized milk from infected cows as a possible infection source and thus provides the necessary data for recommendations on pasteurization of milk fed to calves. In my opinion, this an important paper for the field and the data provided mostly support the conclusions drawn (with an exception for the histology part). However, the presentation and description of the data should be improved. In my opinion, the results section needs to be revised substantially. Currently, it is just a list of results without much explanation of what the authors wanted to test and what was done. This makes it difficult to follow. Moreover, the figures are not easy to understand either. Please see the specific points below:
- The abstract includes a very detailed list of all experiments done. This part (l. 39-57) should be summarized more concisely.
- L. 191: The results section should start with a few sentences saying what the authors wanted to test and how they did that, rather than with the first results description.
- Throughout the results section the authors should explain what they aimed to test and what their experimental procedure was (in short).
- Figure 1: The authors should explain and label better what the scores mean, e.g. what does a score of 2 for “feces” mean?
- In my opinion, fig. 2 is very difficult to understand. Can the authors plot Ct and number of positives separately to make it clearer?
- Fig. 3-7: In order to draw strong conclusions from the histology, the data should include an uninfected control. While it might be clear for a veterinarian that these changes observed are a sign of disease it still needs the uninfected control to demonstrate that.

Reviewer #3

(Remarks to the Author)
The manuscript by Sarlo, Davila, et al., entitled "Susceptibility of Calves Fed Unpasteurized Milk from Cows Experimentally Infected with Highly Pathogenic Avian Influenza H5N1," addresses the impact of feeding unpasteurized milk from HPAIV H5N1-infected cows to calves.
This is an interesting topic that could be relevant in practice. The results showed that calves (4 of 4) could be infected by unpasteurized, antibody-free milk, but only very mild symptoms were observed, and replication efficiency in the animals was also very low (Ct values >>30) and very short (max 4 days in nasal swabs).

Similar results have been published from other inoculation experiments with HPAIV H5N1 in cell culture material. Inoculation through milk shows no significant differences.
If neutralizing antibodies are present in the milk, infection does not occur in all calves (1 out of 4). Other groups have reported neutralization of HPAIV H5N1 in milk by antibodies, whereby virus cultivation on cell cultures was no longer possible. These in vitro results were confirmed in vivo in the present study.
Overall, the results align with previous reports and experiments on the oro-nasal infection of calves with HPAIV H5N1.

However, the hypothesis that calves can carry the infection to new herds is not very plausible and not proven by the study. First, no transmission has been demonstrated. Second, the HPAIV H5 viruses must reach the udder. An infection via transmission from calves appears highly unlikely and has not yet been proven and especially crucial transmission data are missing.

And unfortunately, the discussion is not reflecting any of these points (comparison with other inoculation studies, very mild clinical signs and very low lever replication, missing data concerning virus isolation, missing animal-to-animal transmission experiments etc.).

Although the study provides interesting partial data, important experimental data is missing, and the available data is not critically discussed. For example, the low-level replication, the missing seroconversion in one calf, and the few single-cell stainings in pathohistology are not addressed.

List of specific points:

Line 22: The hypothesis that calves could be a source for farm-to-farm transmission is highly speculative and not further proven. Especially epidemiological data from affected farms are not provided for support (see above)

Line 52: Virus isolation is not mentioned, but would be important.

Line 56: The calve in the group with milk with neutralizing antibodies showed low levels of viral RNA, but seroconversion could not be studied. RNA contamination via the milk has to be considered. Partial protection is therefore a critical term, since the zero-data for the RNA-positive calve are missing.

Line 58: The recommendation should have been part of standard control procedures in affected farms. It is difficult to understand that such an obvious recommendation took over a year to be made. Additionally, one more aspect is missing: The risk of HPAIV H5N1 further adapting to the respiratory tract of bovines and the risk of a new respiratory disease in cattle.

Line 74: Information is very much outdated and have to be updated

Lines 131ff: Virus isolation is missing and data have to be provided to allow conclusions about the infectivity of the collected samples.

Lines 143ff: There is very good H5 antibody ELISAs available which are highly sensitive and specific. Unfortunately these valuable data are missing.

Lines 191 ff: The HPAIV H5N1 titers in the used milk is missing.

Figure 3: The x-axis description has to be 40 minus Ct. The average CT is not shown!

Figure 6: There are only single cells positive. This could be debris collected by immune cells and is not a prove of active replication in these organs.

Figure 7: The changes are at a minimum level and only a few single „stained" cells are not very convincing.

Lines 334ff: H5-ELISA-data are missing. It is also recommended to use the serum neutralization assay as a further confirmatory assay.

Lines 354 to 368: All results (clinical score, PCR, patho-histology) are very minor and only seen for a very short time. CT-values are always above 31 and in most cases even higher. However, these observations are not well reflected in the discussion. Furthermore, the important virus isolation data are missing.

Line 371ff: That the neutralization is not preventing transmission in one animal is not well proven. Seroconversion could not be shown since the animal was euthanized at day 6. Therefore, the conclusion provided is highly speculative.

Lines 393 ff: Also in this study, the ct values are very high and the replication is on a very low level. This is consistent with other studies with calves, which were not mentioned here. Also here the very low level of changes and virus replication is not reflected in the discussion.

Lines 410 and 411: This conclusion is not supported by the data and is highly speculative. Transmission data are missing, virus isolation is missing and field data are mussing. The data do not allow this kind of conclusion (despite the fact that virus positive raw milk should not be fed due to the risk of a possible virus adaptation to cattle).

Extended data 1: The viral titers in the used milk are missing. CT values are not enough to allow valid conclusions.

Version 1:

Reviewer comments:

Reviewer #1

(Remarks to the Author)
In the revised version, the authors have addressed some of my concerns. I still consider the findings important and of interest to the field and some of the figures and descriptions (e.g. fig. 2) have been improved. However, I still think that presentation and description of the findings, in particular the abstract and the results section, are not amenable to the broad audience of the journal.
Specific points:
- In their rebuttal, the authors claim that the abstract is now more concise but I do not agree. It is still mainly a list of very detailed results that are not summarized concisely.
- L.64 should be updated.
- L. 185-187: The description of what has been done and why is still not clear: "RT-PCR detection of influenza A virus reported as Ct values and virus titers of the pooled milk with virus (Group One) and milk with virus and antibody (Group Two) from experimentally inoculated cows from intramammary inoculated cows fed to calves." It is unclear to me what this means. This needs to be worded more clearly.
- The different sections of the results part have titles based on the method used. I would find it more useful if the sections had titles according to the question addressed or the result obtained.
- L. 389: What do the authors mean with "As we reduced, …, the transmission…"? Are they referring to the antibodies? It sounds like the authors applied some method to prevent transmission but probably they are just referring to the antibodies.
- The authors say they now included histology from an uninfected cow but I could only find the figure legend of Extended Data Fig. 5, not the figure.

Reviewer #3

(Remarks to the Author)
Many of the reviewers' suggestions have been implemented. In particular, several text passages that were not supported by the data were revised. In addition, the authors have mentioned new data points (e.g., virus isolation and titration of the inoculation material).
However, several issues remain unclear and require clarification and/or further improvement:
1. Change marking / highlights: The yellow highlights in the revised manuscript are incomplete. In particular, deletions and some newly added text/data were only visible in the highlighted word file within the ZIP folder. This is unfortunate and made it difficult to track and evaluate the revisions.
2. Table 1 remains unclear. Why are high virus titers present in milk at day 0 and the following days and what is the reason for the titer differences? What exactly was measured, and why is there no neutralization effect despite detectable antibodies in group 2? The table needs to be checked and clarified, and the table legend/description should be rewritten for clarity (e.g., inconsistent wording such as "pooled milk" vs. "milk," etc.).
3. Table 2 (fever definition): A fever threshold of 39.5°C is often recommended for cattle. Using 39.0°C is likely not optimal. Please consult the literature and justify the chosen cut-off; 39.5°C might be a more reasonable definition.
4. Lines ~313 ff. (Ct values and virus isolation): All reported swab Ct values appear to be >30. In the rebuttal letter, Ct 30 was stated as the threshold for attempting virus isolation. The rebuttal further states that virus isolation in embryonated chicken eggs was performed with samples < Ct 30 and that this was successful for swabs, but the corresponding new data are not shown. Please reconcile the abstract (around line 55), the main text, and the rebuttal letter, and provide the missing isolation results (or correct the statements). Also the extended data (2 and) are not showing virus isolation from swabs, but for the tissues. But in the table it remained unclear what is the exact CT value (red only says CT < 35).
5. Discussion (lines ~454–455; isolation results and sample selection): Virus isolation is again mentioned, but the underlying data are neither presented nor clearly described in the manuscript. "Extended data 3" is cited, yet the relevant isolation results are not shown there either. Overall, this is confusing. The selection criteria for isolation attempts and the isolation outcomes should be clearly reported (including which samples were chosen and why, and the success rate). Additionally, the scheme marking samples with Ct <35 as "red/most positive" is unusual, as Ct 35 typically corresponds to very low genome copy numbers close to the detection limit and does not indicate a high viral load.
6. Strength of detection signals: As noted in the first review, the virus detection data in the inoculated calves are weak overall, with many results close to the limit of detection. Also the argument of staining within the nucleus is not very convincing, since there is so few positive signals overall.
7. Discussion revision: The discussion has only very minor changes, and key points raised by the reviewer were not adequately addressed. The discussion should be reworked to clearly state that most detection signals are near the limit of detection, and that even feeding extremely high titers (10^8 per mL milk) did not lead to clear clinical signs or replication levels clearly above the detection limit in most samples. As a further example is the statement from lines 467 to 470 confusing („This work also demonstrates that the presence of neutralizing antibody in the milk can impact H5N1 transmission, as we reduced, but did not completely prevent, the transmission of H5N1 genotype B3.13 to calves. No clinical signs of disease nor seroconversion were observed in these calves (Group Two)." Why do the authors interpret these data as evidence that infection was not fully prevented? The sporadic, low-level RNA detection in only a few samples, together

with the absence of seroconversion, rather supports the conclusion that the animals were protected from productive infection. At a minimum, the manuscript should clearly explain why near-limit-of-detection Ct signals in a few samples are biologically meaningful and reconcile this interpretation with the lack of serological evidence for infection.

Version 2:

Reviewer comments:

Reviewer #1

(Remarks to the Author)
The authors have addressed all remaining points.

Reviewer #3

(Remarks to the Author)
The manuscript has further improved in this re-revision. I have only two minor comments that should be addressed:

1. Please delete the duplication in the sentence “For Group 1 calves were fed milk from experimentally two experimentally…” in line 186.

2. In the legend to Figure 1, the word “milk” is missing. The sentence should read: “Figure 1. A) Clinical observations of Group One calves fed milk containing virus.”

Open Access This Peer Review File is licensed under a Creative Commons Attribution 4.0 International License, which permits use, sharing, adaptation, distribution and reproduction in any medium or format, as long as you give appropriate credit to the original author(s) and the source, provide a link to the Creative Commons license, and indicate if changes were made.
In cases where reviewers are anonymous, credit should be given to 'Anonymous Referee' and the source.
The images or other third party material in this Peer Review File are included in the article’s Creative Commons license, unless indicated otherwise in a credit line to the material. If material is not included in the article’s Creative Commons license and your intended use is not permitted by statutory regulation or exceeds the permitted use, you will need to obtain permission directly from the copyright holder.
To view a copy of this license, visit https://creativecommons.org/licenses/by/4.0/

Nature Comms Reviewer Comments

Reviewer #1 (Remarks to the Author):

The study by Sarlo Davila et al. tested the susceptibility of calves to infection with highly pathogenic avian influenza H5N1via feeding with milk from infected cows. Given the continuing problems caused by highly pathogenic avian influenza H5N1 in dairy cattle in the US this is an important question to answer. Calves fed infected milk without antibodies showed clinical signs including nasal discharge, mild fever, loose stool and slightly increased respiratory effort for 5-6 days. Virus could be detected in multiple tissues and calves seroconverted. Calves fed infected milk that also contained maternal H5-specific antibodies did not develop clinical symptoms and only 1/4 calves had detectable virus and no seroconversion was detected.
The study sheds light on calves fed with unpasteurized milk from infected cows as a possible infection source and thus provides the necessary data for recommendations on pasteurization of milk fed to calves. In my opinion, this an important paper for the field and the data provided mostly support the conclusions drawn (with an exception for the histology part). However, the presentation and description of the data should be improved. In my opinion, the results section needs to be revised substantially. Currently, it is just a list of results without much explanation of what the authors wanted to test and what was done. This makes it difficult to follow. Moreover, the figures are not easy to understand either. Please see the specific points below:
- The abstract includes a very detailed list of all experiments done. This part (l. 39-57) should be summarized more concisely.
Thank you for this suggestion, the experiments have been summarized more concisely.

- L. 191: The results section should start with a few sentences saying what the authors wanted to test and how they did that, rather than with the first results description.
- Throughout the results section the authors should explain what they aimed to test and what their experimental procedure was (in short). Thank you for this suggestion, a brief explanation of what we wanted to test and how it was tested was added to the results section.

- Figure 1: The authors should explain and label better what the scores mean, e.g. what does a score of 2 for “feces” mean? Thank you for this suggestion, a further explanation has been added and the axis labels have been updated.

- In my opinion, fig. 2 is very difficult to understand. Can the authors plot Ct and number of positives separately to make it clearer? Thank you for this suggestion, Ct and number of positive have been plotted separately.

- Fig. 3-7: In order to draw strong conclusions from the histology, the data should include an uninfected control. While it might be clear for a veterinarian that these changes observed are a sign of disease it still needs the uninfected control to demonstrate that.

Thank you for your comment, an Extended Data Figure 5 is now included and consists of photomicrographs of a H&E lung section and influenza A virus nucleoprotein immunohistochemistry of the lung, parotid lymph node, mandibular lymph node, tracheobronchial lymph node, retropharyngeal lymph node and pharyngeal tonsil from an uninfected control. Additionally, the figure legends of Figure 4 and Extended Data Figure 1 have been revised to more clearly illustrate the macroscopically unremarkable tissue in inoculated calves. The legend of Figure 4 now reads, "Lung lesions and lymphadenomegaly of Group One calves fed milk containing virus. Minimal multifocal obstructive atelectasis (arrowheads) consistent with influenza A virus at 6 dpi in calf 2410 (A) and 2414 (B). A majority of the lung in both calves was macroscopically unremarkable and not denoted. B) Lymphadenomegaly (arrows) of the mediastinal lymph node in calf 2414." The legend of Extended Data Figure 1 now reads, "Extended Data Figure 1. Minimal multifocal obstructive atelectasis (arrowheads) consistent with influenza A virus at 6 dpi in calf 2411. A majority of the lung is macroscopically unremarkable and not denoted."

Reviewer #3 (Remarks to the Author):

The manuscript by Sarlo, Davila, et al., entitled "Susceptibility of Calves Fed Unpasteurized Milk from Cows Experimentally Infected with Highly Pathogenic Avian Influenza H5N1," addresses the impact of feeding unpasteurized milk from HPAIV H5N1-infected cows to calves.
This is an interesting topic that could be relevant in practice. The results showed that calves (4 of 4) could be infected by unpasteurized, antibody-free milk, but only very mild symptoms were observed, and replication efficiency in the animals was also very low (Ct values >>30) and very short (max 4 days in nasal swabs).

Similar results have been published from other inoculation experiments with HPAIV H5N1 in cell culture material. Inoculation through milk shows no significant differences.
If neutralizing antibodies are present in the milk, infection does not occur in all calves (1 out of 4). Other groups have reported neutralization of HPAIV H5N1 in milk by antibodies, whereby virus cultivation on cell cultures was no longer possible. These in vitro results were confirmed in vivo in the present study.
Overall, the results align with previous reports and experiments on the oro-nasal infection of calves with HPAIV H5N1.

However, the hypothesis that calves can carry the infection to new herds is not very plausible and not proven by the study. First, no transmission has been demonstrated. Second, the HPAIV H5 viruses must reach the udder. An infection via transmission from calves appears highly unlikely and has not yet been proven and especially crucial

transmission data are missing.

And unfortunately, the discussion is not reflecting any of these points (comparison with other inoculation studies, very mild clinical signs and very low lever replication, missing data concerning virus isolation, missing animal-to-animal transmission experiments etc.).

Although the study provides interesting partial data, important experimental data is missing, and the available data is not critically discussed. For example, the low-level replication, the missing seroconversion in one calf, and the few single-cell stainings in pathohistology are not addressed.

List of specific points:

Line 22: The hypothesis that calves could be a source for farm-to-farm transmission is highly speculative and not further proven. Especially epidemiological data from affected farms are not provided for support (see above) Thank you for this suggestion. The statement revised to line 58 stating "the risk of calves as a source of farm to farm transmission has not yet been evaluated".

Line 52: Virus isolation is not mentioned, but would be important.

Thank you for this suggestion, virus isolation has been performed and the results included in Extended Data 3.

Line 56: The calve in the group with milk with neutralizing antibodies showed low levels of viral RNA, but seroconversion could not be studied. RNA contamination via the milk has to be considered. Partial protection is therefore a critical term, since the zero-data for the RNA-positive calve are missing.
Thank you this has been updated to at least partially protected.

Line 58: The recommendation should have been part of standard control procedures in affected farms. It is difficult to understand that such an obvious recommendation took over a year to be made. Additionally, one more aspect is missing: The risk of HPAIV H5N1 further adapting to the respiratory tract of bovines and the risk of a new respiratory disease in cattle.
Thank you for this suggestion, this has been addressed " as H5N1 may further adapt to the bovine respiratory tract and the risk of calves as a source of farm to farm transmission has not yet been evaluated"
Line 74: Information is very much outdated and have to be updated. Thank you this has been updated

Lines 131ff: Virus isolation is missing and data have to be provided to allow conclusions about the infectivity of the collected samples.

Thank you for this suggestion, virus isolation has been performed and the results included

Lines 143ff: There is very good H5 antibody ELISAs available which are highly sensitive and specific. Unfortunately these valuable data are missing. We agree H5 antibody ELISAs are very valuable and the ID Screen® Influenza H5 Antibody Competition 3.0 Multi-Species, Innovative Diagnostics, Grabels, France was used.

Lines 191 ff: The HPAIV H5N1 titers in the used milk is missing.
Thank you for this suggestion, this information has been added to Table1.

Figure 3: The x-axis description has to be 40 minus Ct. The average CT is not shown!

Figure 6: There are only single cells positive. This could be debris collected by immune cells and is not a prove of active replication in these organs.

The authors agree that some of these cells may very well be antigen presenting cells that have phagocytosed IAV NP antigen. However, in panel C of Figure 6 (2410) and panel A and B of Extended Data Figure 2 (Calf 2414), there is nuclear staining as denotes by the arrowhead. Antigen processing does not have a step that occurs in the nucleus. In the context of IAV nucleoprotein (NP) detection in the nucleus of cells and the IAV replication cycle, the presence of NP protein as detected by IHC in the nucleus of cells is consistent with replicating virus. https://doi.org/10.1371/journal.pone.0164146: "As influenza virus replicates inside the nucleus, NP continuously shuttles between the nucleus and the cytoplasm interacting with various host factors to regulate multiple functions during the replication [15]. Viral RNP complex is imported into the nucleus through its interaction with importin α [16], and viral RNA synthesis is regulated by interaction between NP and cellular factors such as BAT1/UAP56, Tat-SF1, MCM complexes [17, 18, 19]. So is export of vRNA into the cytoplasm mediated by NP interaction with CRM1 [20]. Furthermore, NP association with cytoskeletal proteins such as alpha actinin-4 demonstrated to be essential for its translocation into the nucleus [21]." This is also stated in the manuscript Paquette et al. page 14: "Positive NP stain was used to determine if viral replication may be occurring within the nucleus. At 24 h post-inoculation influenza NP was detected in the nucleus (white arrows) of all three cell types." The detection of this protein 6 DPI in the nucleus is most consistent with NP protein performing its role in the virus replication cycle. Different leukocyte subsets are differentially susceptible to IAV infection/replication:

1. Chan, MCW, Lee N, Chan PKS, et al. Intestinal binding of seasonal influenza A viruses to DC-SIGN+ CD68+ cells. Influ. Other. Respir. Viruses. 2013;7:228–230. doi: 10.1111/j.1750-2659.2012.00402.
2. Nichols JE, Mock DJ, Roberts NJ Jr. Use of FITC-labeled influenza virus and flow cytometry to assess binding and internalization of virus by monocytes-macrophages and lymphocytes. Arch. Virol. 1993;130(3-4):441-455. doi: 10.1007/BF01309672.

3. Yen HL, Aldridge JR, Boon AC, et al. Changes in H5N1 influenza virus hemagglutinin receptor binding domain affect systemic spread. Proc. Natl. Acad. Sci. USA. 2009;106(1):286-291. doi: 10.1073/pnas.0811052106.
4. Moltedo B, Li W, Yount JS, et al. Unique type I interferon responses determine the functional fate of migratory lung dendritic cells during influenza virus infection. PloS. Pathog. 2011;7(11):e1002345. doi: 10.1371/journal.ppat.1002345.
5. Helft J, Manicassamy B, Guermonprez P, et al. Cross-presenting CD103+ dendritic cells are protected from influenza virus infection. J. Clin. Invest. 2012;122(11):4037-47. doi: 10.1172/JCI60659.
6. Mock DJ, Frampton MW, Nichols JE, et al. Influenza Virus Infection of Human Lymphocytes Occurs in the Immune Cell Cluster of the Developing Antiviral Response. Viruses. 2018;10(8):420. doi: 10.3390/v10080420.

Figure 7: The changes are at a minimum level and only a few single „stained“ cells are not very convincing.
While the authors acknowledge that the number of cells containing the antigen is limited, there is also nuclear staining observed in a subset of these cells, which is consistent with viral replication. Additionally, the virus was detected by RT-qPCR at 6 DPI. Fresh and fixed samples were collected days after peak infection and three days after the cessation of feeding infected milk. The authors use the word 'suggests' to propose that this may represent an additional site of IAV replication in cattle, and they fully support further investigation at more acute timepoints, as outlined in the Discussion.

Lines 334ff: H5-ELISA-data are missing. It is also recommended to use the serum neutralization assay as a further confirmatory assay. H5 ELISA data are shown in columns 5(6 DPI) and 9 (13 DPI) of Table 3. Hemagglutinin inhibition assays were performed as a further confirmatory assay and results are shown in columns 6 (6 DPI) and 10 (13 DPI) of Table 3.

Lines 354 to 368: All results (clinical score, PCR, patho-histology) are very minor and only seen for a very short time. CT-values are always above 31 and in most cases even higher. However, these observations are not well reflected in the discussion. Furthermore, the important virus isolation data are missing.
Thank you for this suggestion, virus isolation has been performed and the results included

Line 371ff: That the neutralization is not preventing transmission in one animal is not well proven. Seroconversion could not be shown since the animal was euthanized at day 6. Therefore, the conclusion provided is highly speculative. Thank you for this suggestion, this has been addressed as “The lack of seroconversion supports the observation that transmission was prevented in the 2 calves that survived to 13 DPI, but additional timepoints are warranted in future studies.”.

Lines 393 ff: Also in this study, the ct values are very high and the replication is on a very low level. This is consistent with other studies with calves, which were not mentioned here. Also here the very low level of changes and virus replication is not reflected in the discussion. Thank you for this suggestion, this has been addressed as "Viral replication was at a low level but virus was isolated from nasal swabs and tissues samples, as shown in Extended Data 3."

Lines 410 and 411: This conclusion is not supported by the data and is highly speculative. Transmission data are missing, virus isolation is missing and field data are mussing. The data do not allow this kind of conclusion (despite the fact that virus positive raw milk should not be fed due to the risk of a possible virus adaptation to cattle). Thank you for this suggestion, this has been addressed as "warrant future studies investigating calf to calf transmission and the adaptation of H5N1 to the bovine respiratory tract."

Extended data 1: The viral titers in the used milk are missing. CT values are not enough to allow valid conclusions.
Thank you for this suggestion, this has been moved from the extended data to table 1 and the titers have been included.

Reviewer #1 (Remarks to the Author):

In the revised version, the authors have addressed some of my concerns. I still consider the findings important and of interest to the field and some of the figures and descriptions (e.g. fig. 2) have been improved. However, I still think that presentation and description of the findings, in particular the abstract and the results section, are not amenable to the broad audience of the journal.
Specific points:
- In their rebuttal, the authors claim that the abstract is now more concise but I do not agree. It is still mainly a list of very detailed results that are not summarized concisely.

Thank you for this suggestion, the abstract has been summarized more concisely.

- L.64 should be updated.
Line 64 has been updated

- L. 185-187: The description of what has been done and why is still not clear: "RT-PCR detection of influenza A virus reported as Ct values and virus titers of the pooled milk with virus (Group One) and milk with virus and antibody (Group Two) from experimentally inoculated cows from intramammary inoculated cows fed to calves." It is unclear to me what this means. This needs to be worded more clearly.

Thank you for this suggestion, this has been worded more clearly.

- The different sections of the results part have titles based on the method used. I would find it more useful if the sections had titles according to the question addressed or the result obtained.

Thank you for this suggestion. The sections have been organized by result obtained and are now "**Virus and neutralizing antibodies in milk fed to calves", "Clinical signs of infection in calves", "Virus detection", and "Serological responses"**

- L. 389: What do the authors mean with "As we reduced, ..., the transmission..."? Are they referring to the antibodies? It sounds like the authors applied some method to prevent transmission but probably they are just referring to the antibodies.
Thank you for this suggestion. This has been reworded to "antibodies prevented" for clarity.

- The authors say they now included histology from an uninfected cow but I could only find the figure legend of Extended Data Fig. 5, not the figure.
We apologize for this oversight, the Figure has been included.

Reviewer #3 (Remarks to the Author):

Many of the reviewers' suggestions have been implemented. In particular, several text passages that were not supported by the data were revised. In addition, the authors have mentioned new data points (e.g., virus isolation and titration of the inoculation material). However, several issues remain unclear and require clarification and/or further improvement:

1. Change marking / highlights: The yellow highlights in the revised manuscript are incomplete. In particular, deletions and some newly added text/data were only visible in the highlighted word file within the ZIP folder. This is unfortunate and made it difficult to track and evaluate the revisions.

We apologize for the difficulty caused. We have used the track changes function of word to facilitate easier tracking and evaluation of the revisions.

2. Table 1 remains unclear. Why are high virus titers present in milk at day 0 and the following days and what is the reason for the titer differences? What exactly was measured, and why is there no neutralization effect despite detectable antibodies in group 2? The table needs to be checked and clarified, and the table legend/description should be rewritten for clarity (e.g., inconsistent wording such as "pooled milk" vs. "milk," etc.).

Thank you for this suggestion. This table has been checked for consistency and the variation in virus titers due to DPI of cows from which the milk was collected has been clarified.

3. Table 2 (fever definition): A fever threshold of 39.5°C is often recommended for cattle. Using 39.0°C is likely not optimal. Please consult the literature and justify the chosen cut-off; 39.5°C might be a more reasonable definition.

Thank you for this suggestion, the fever threshold has been adjusted to 39.5°C.

4. Lines ~313 ff. (Ct values and virus isolation): All reported swab Ct values appear to be >30. In the rebuttal letter, Ct 30 was stated as the threshold for attempting virus isolation. The rebuttal further states that virus isolation in embryonated chicken eggs was performed with samples < Ct 30 and that this was successful for swabs, but the corresponding new data are not shown. Please reconcile the abstract (around line 55), the main text, and the rebuttal letter, and provide the missing isolation results (or correct the statements). Also the extended data (2 and) are not showing virus isolation from swabs, but for the tissues. But in the table it remained unclear what is the exact CT value (red only says CT < 35).

Thank you for this suggestion. We have clarified that virus isolation was attempted for all nasal swabs and tissues with Ct < 30. Extended data tables have been updated to show Ct values for all tissues and tissues with successful virus isolation are not highlighted in red and all other color schemes have been removed. Figure 2 has been edited, and 2B now shows the number of virus isolation positive nasal swabs from group one. 2D has been removed as there were not virus isolation positive nasal swabs from Group 2.

5. Discussion (lines ~454–455; isolation results and sample selection): Virus isolation is again mentioned, but the underlying data are neither presented nor clearly described in the manuscript. "Extended data 3" is cited, yet the relevant isolation results are not shown there either. Overall, this is confusing. The selection criteria for isolation attempts and the isolation outcomes should be clearly reported (including which samples were chosen and why, and the success rate). Additionally, the scheme marking samples with Ct <35 as "red/most positive" is unusual, as Ct 35 typically corresponds to very low genome copy numbers close to the detection limit and does not indicate a high viral load.

Thank you for this suggestion. Extended Data 2 has been modified for clarity. The Ct values for all samples are now shown without a color scheme and only samples with positive virus isolation are shown in red.

6. Strength of detection signals: As noted in the first review, the virus detection data in the inoculated calves are weak overall, with many results close to the limit of detection. Also the argument of staining within the nucleus is not very convincing, since there is so few positive signals overall.

Thank you for this suggestion. We feel that the inclusion of virus isolation data strengthens this argument by providing an additional positive signal.

7. Discussion revision: The discussion has only very minor changes, and key points raised by the reviewer were not adequately addressed. The discussion should be reworked to clearly state that most detection signals are near the limit of detection, and that even feeding extremely high titers (10^8 per mL milk) did not lead to clear clinical signs or replication levels clearly above the detection limit in most samples. As a further example is the statement from lines 467 to 470 confusing („This work also demonstrates that the presence of neutralizing antibody in the milk can impact H5N1 transmission, as we reduced, but did not completely prevent, the transmission of H5N1 genotype B3.13 to calves. No clinical signs of disease nor seroconversion were observed in these calves (Group Two)." Why do the authors interpret these data as evidence that infection was not fully prevented? The sporadic, low-level RNA detection in only a few samples, together with the absence of seroconversion, rather supports the conclusion that the animals were protected from productive infection. At a minimum, the manuscript should clearly explain why near-limit-of-detection Ct signals in a few samples are biologically meaningful and reconcile this interpretation with the lack of serological evidence for infection.

Thank you for this suggestion. Upon further review, the authors agree that animals were protected against productive infection and the discussion has been amended. The discussion has also be amended to note that many detection signals were near the limit of detection in the calves that did become infected.

Reviewer #1 (Remarks to the Author):

The authors have addressed all remaining points.

Reviewer #3 (Remarks to the Author):

The manuscript has further improved in this re-revision. I have only two minor comments that should be addressed:

1. Please delete the duplication in the sentence “For Group 1 calves were fed milk from experimentally two experimentally...” in line 186.
Thank you, the duplication has been deleted.

2. In the legend to Figure 1, the word “milk” is missing. The sentence should read: “Figure 1. A) Clinical observations of Group One calves fed milk containing virus.”

Thank you, this has been corrected.
